# Pesticides and Trace Element Residues in Honey from Northern Croatia

**DOI:** 10.3390/foods15091502

**Published:** 2026-04-25

**Authors:** Damir Pavliček, Marija Sedak, Nina Bilandžić, Ivana Varenina, Ivana Tlak Gajger, Anton Gradišek, Mariša Ratajec, Maja Đokić

**Affiliations:** 1 Laboratory for Microbiology and Analytical Chemistry, Croatian Veterinary Institute Zagreb, Department Križevci, Zakmardijeva 10, 48260 Križevci, Croatia; 2 Laboratory for Residue Control, Department of Veterinary Public Health, Croatian Veterinary Institute, Savska cesta 143, 10000 Zagreb, Croatia; sedak@veinst.hr (M.S.); bilandzic@veinst.hr (N.B.); kurtes@veinst.hr (I.V.); dokic@veinst.hr (M.Đ.); 3 Department for Biology and Pathology of Fish and Bees, Faculty of Veterinary Medicine, University of Zagreb, Heinzelova 55, 10000 Zagreb, Croatia; itlak@vef.unizg.hr; 4 Department of Intelligent Systems, Jožef Stefan Institute, Jamova Cesta 39, SI-1000 Ljubljana, Slovenia; anton.gradisek@ijs.si; 5 Faculty of Electrical Engineering, University of Ljubljana, Tržaška Cesta 25, SI-1000 Ljubljana, Slovenia; ratajec.marisa@gmail.com

**Keywords:** pesticides, trace elements, residues, honey, comb honey, risk assessment

## Abstract

The rapid translocation of pesticide and metal residues in the environment and their entry into the food chain pose a significant risk to human health. Given the high global consumption of honey, quality control emphasizes the need for continuous monitoring and risk assessment. To evaluate contamination levels in honey from northern Croatia, a region with intensive agricultural land use, 38 comb honey and 22 extracted honey samples were collected by purposive one-time sampling in June 2023. These samples were analyzed for 190 pesticides using liquid chromatography–tandem mass spectrometry (LC-MS/MS) and gas chromatography–tandem mass spectrometry (GC-MS/MS), and for 17 trace metal(loid)s using inductively coupled plasma mass spectrometry (ICP-MS). The highest detection frequencies were observed for fipronil-sulfone, trifloxystrobin, and coumaphos in comb honey, and for *N*-(2,4-dimethylphenyl)-formamide (DMF) and *N*-(2,4-dimethylphenyl)-*N*′-methylformamidine (DPMF) in extracted honey. Glyphosate was the only pesticide to exceed the European Union (EU) maximum residue level (MRL) of 0.05 mg/kg in three honey samples. Elemental analysis quantified most target metals, with aluminum (Al), copper (Cu), iron (Fe), manganese (Mn), nickel (Ni) and zinc (Zn) being the most abundant, while silver (Ag), arsenic (As), and selenium (Se) were not detected in this study. None of the samples contained lead (Pb) above the regulatory limit for honey established in the EU (0.1 mg/kg). To ensure food safety, further efforts are required to assess the health risks associated with exposure to these contaminants through consumption of the evaluated food.

## 1. Introduction

Honeybee products are valued worldwide for their nutritional, medicinal, and economic significance. Honey consists mainly of sugars but also contains vitamins, minerals, enzymes, and bioactive compounds that contribute to its recognized antioxidant, antimicrobial, and anti-inflammatory properties [1]. Due to these qualities, honey is widely consumed by diverse populations of different ages and is commonly regarded as a natural, safe food. Comb honey, honey stored by bees within the wax cells of the honeycomb, offers further nutritional complexity by including beeswax and trace components from hive matrices, which may reflect the environmental exposures experienced by worker bees [2].

Honey bees (*Apis mellifera* L.) forage up to several kilometers from the hive, collecting nectar, pollen, water, and resinous plant exudates (propolis) from their environment. During foraging, worker bees inevitably encounter environmental contaminants, including plant protection products (PPPs) such as insecticides, herbicides, and fungicides used in agriculture, as well as toxic elements present in soil, water, and air [3]. Once these contaminants enter the hive, they can be incorporated into honey and other honeybee products, including beeswax and comb honey. Pesticide residues from various chemical classes with different physicochemical properties can be detected in honey, sometimes at concentrations that approach or exceed established MRLs [4,5].

The complexity of the honey matrix and the diversity of potential environmental contaminants present significant analytical challenges. Multi-residue analytical strategies, such as QuEChERS extraction followed by LC-MS/MS or GC-MS/MS, are commonly used to quantify trace levels of pesticides in honey with high sensitivity and specificity. These methods often cover hundreds of analytes across multiple chemical classes [6]. While most analyses have focused on extracted honey, comb honey may accumulate contaminants differently due to the additional contributions of beeswax and interactions with hive matrices. Beeswax, with its lipid-rich composition, can concentrate more lipophilic pesticide residues than honey, raising concerns about the cumulative contamination potential of comb honey and beeswax [7]. Research comparing honey and beeswax samples from Israeli hives found that more lipophilic pesticides tended to partition into beeswax, while certain pesticides and trace elements were detectable in both honey and wax matrices. As beeswax binds and retains numerous xenobiotics, this may have implications for human exposure if comb honey is consumed directly with the wax components [8].

In addition to pesticides, heavy metals and metalloid contaminants constitute another major class of environmental pollutants that can accumulate in honeybee products. Metal(loid)s such as As, cadmium (Cd), Pb and mercury (Hg) enter ecosystems through natural processes such as soil erosion and volcanic activity, as well as anthropogenic sources including industrial emissions, mining, traffic, and intensive agriculture [9]. These elements can be absorbed by flowering plants and subsequently transferred into nectar and pollen, exposing bees to these contaminants during foraging. Honey and comb honey therefore serve not only as food products but also as bioindicators of environmental contamination, reflecting the cumulative impact of pollutants in the landscape surrounding apiaries.

Although many authors have reported toxicological concerns about negative effects on human health from consuming honey containing pesticide residues or metalloids [5,10,11], their synergistic effects have, to our knowledge, not yet been addressed. However, the importance of combined chemical exposure is well recognized in current toxicological risk assessment frameworks [12,13]. Mixture toxicity is a critical aspect of risk assessment for environmental contaminants, as humans are usually exposed to multiple chemicals simultaneously rather than to individual substances. Reliable evaluation of their potential adverse health effects requires extensive and comprehensive data, but understanding the interactions among different compounds remains challenging due to methodological and technical limitations [14]. Therefore, the available literature on honey mainly presents occurrence data and separate or cumulative risk estimates, while confirmed mixture-synergy data for this matrix are generally lacking.

The analytical determination of pesticides and trace elements in honey and comb honey is important for scientific and regulatory purposes, including food safety assessment, environmental biomonitoring, and apicultural health research. Accurate quantification of these contaminants relies on reliable analytical methods, such as inductively coupled plasma mass spectrometry (ICP-MS) for metal analysis and chromatographic techniques for multi-class pesticide residue determination, calibrated against internationally recognized standards and maximum residue limits where available. These methods enable sensitive detection of trace contaminants, supporting risk assessment and verification of regulatory compliance.

In this study, we aim to determine the residue concentrations of selected compounds from the acaricide, fungicide, herbicide, insecticide, and nematicide classes of pesticides, as well as trace elements, in honey and comb honey samples from a geographic region in Croatia that has recently become a focal point for mass colony collapse incident. By comparing contaminant profiles between these related but distinct matrices and evaluating the data against established safety benchmarks, we aim to improve understanding of environmental exposure pathways, risks to human health, and implications for sustainable apicultural practices and food safety.

## 2. Materials and Methods

### 2.1. Sample Collection and Storage

**Figure 1:**
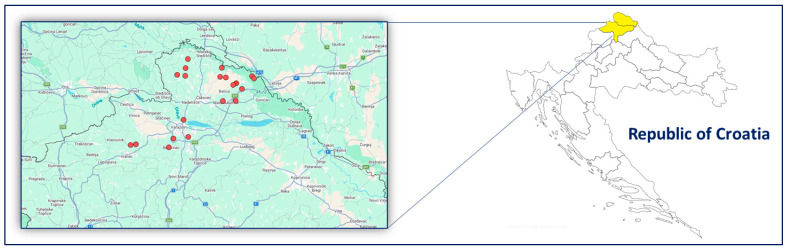
Sampling sites in northern Croatia (Međimurje and Varaždin County).

Honey sampling was carried out in June 2023, immediately following the Acacia (*Robinia pseudoacacia*) honey flow, at the transition to the period of intensive treatment of surrounding crops (e.g., oilseed rape, orchards). Collecting honey at this time captures residues accumulated during the early season, shortly after pesticide applications in adjacent agricultural areas, and provides an integrated measure of exposure over a critical period. In total, 38 samples of comb honey and 22 samples of freshly extracted honey from honeycombs were collected at 20 apiary sites in Međimurje and Varaždin County (Figure 1). Apiaries were selected by purposive sampling, focusing on areas with a documented history of repeated bee mortality incidents and intensive agricultural land use. Sampling was performed by beekeepers following a working protocol specifying all details. After placing the collected samples (comb honey: three pieces of honeycomb, each 10 cm^2^, containing ripe honey in a PE zip lock bag; honey: 200 g in a PE/PPE bottle) in a portable refrigerator (2–8 °C), they were transported to the laboratory and stored at a temperature of −18 °C until analysis. Honey from the collected honeycombs was previously extracted by placing it on a flat, clean surface. The surface layer was removed with a fork or spoon, and wooden or glass utensils were used to press and push the honey from the honeycomb cells into a clean vial.

### 2.2. Pesticide Analyses

#### 2.2.1. Chemicals and Standards

Acetonitrile (LC-MS grade, ≥99.9%), cyclohexane (HPLC grade, ≥99.7%), ethyl acetate (HPLC grade, ≥99.7%), *N,N*-Dimethylformamide (DMF_sol_, HPLC grade, ≥99.9%), dichloromethane (DCM, HPLC grade, ≥99.8%) and acetone (HPLC grade, ≥99.8%) were purchased from Chromasolv (Honeywell Specialty Chemicals, Seelze, Germany). Methanol (LC-MS grade, ≥99.9%) and formic acid (LC-MS grade, 98–100%) were purchased from Sigma-Aldrich (Steinheim, Germany). Ultrapure water was produced by the Milli-Q system (Millipore, Bedford, MA, USA). The QuEChERS kit used for the extraction step was Bekolut Citrate Kit 1 (LCTech GmbH, Dorfen, Germany), containing a 50 mL extraction tube with 4 g magnesium sulfate (MgSO_4_), 1 g sodium chloride (NaCl), 1 g trisodium citrate dihydrate (TSC) and 0.5 g disodium hydrogen citrate sesquihydrate (DHC). For the clean-up step, we used a Bekolut PSA-Kit-02 (LCTech GmbH, Dorfen, Germany) which consists of a 15 mL tube containing 900 mg of MgSO_4_ and 150 mg primary–secondary amine (PSA) sorbent. For the modified Swedish ethyl acetate (SweEt) method [15] and extraction, we used a mixture of sodium sulphate anhydrous (Na_2_SO_4_), TSC and DHC (all reagent grade, ≥99.0%), purchased from Sigma-Aldrich (Steinheim, Germany), PSA was purchased from Macherey-Nagel GmbH & Co. KG (Düren, Germany) while NaCl (analytical grade, ≥99.5%) was purchased from Supelco, Merck KGaA (Darmstadt, Germany).

High-purity pesticide analytical standards and isotopically labeled standards were purchased from Dr. Ehrenstorfer (Augsburg, Germany), Sigma-Aldrich (Steinheim, Germany), Supelco (Bellefonte, PA, USA), HPC Standards GmbH (Cunnersdorf, Germany), Qpp-Lab (Castellana Grotte, Italy), and Institute of Industrial Organic Chemistry (Warsaw, Poland).

For pesticides, individual standard stock solutions (1 mg/mL) were prepared in acetone and DMF_sol_, and stored in amber glass bottles at −18 °C. The final working solutions (10 µg/mL, 1 µg/mL) were prepared by mixing appropriate volumes of each standard stock solutions in acetone and stored in amber glass bottles at 4 °C. Individual internal standard (IS) stock solutions were prepared at a concentration of 1 mg/mL in acetone and stored in amber glass bottles at −18 °C. Working solutions of internal standard mixtures were prepared at a concentration of 1 µg/mL for each compound: one mixture containing Amitraz-d12, Dichlorprop-d6 and Carbendazim-d3, and another one containing Mirex and PCB 209. The working solutions were stored in amber glass bottles at 4 °C.

For the highly polar pesticides glyphosate, *N*-acetyl-AMPA, glufosinate and 3-methylphosphinicopropionic acid (MPPA), individual standard stock solutions (1 mg/mL) were prepared in a water/acetonitrile (9:1 *v*/*v*) mixture. The stock solution was further diluted with the same solvent to a concentration of 10 µg/mL, which was used in the procedure. The working solution of the internal standards (glyphosate-13C2,15N, glufosinate-D3, and MPPA-D3) was prepared in the same way at a concentration of 20 µg/mL and stored in amber glass bottles at 5 ± 3 °C for seven months.

#### 2.2.2. Sample Preparation

For the multiresidue analysis of pesticides in honey and comb honey, the SweET method was applied as the sample preparation procedure to efficiently extract GC-amenable compounds. The QuEChERS method, according to Standard HRN EN 15662:2018 [16], was used with slight modifications. This procedure served as the sample preparation step for GC-MS/MS analysis. A 5 ± 0.1 g portion of the sample was weighed in a 50 mL centrifuge tube and spiked with appropriate amounts of pesticides in working solutions and a mixture of internal standards (Mirex, PCB 209). Water (5 mL) and a cyclohexane/ethyl acetate mixture (10 mL) were added to the sample, which was then shaken for 10 min. Next, 4 g of Na_2_SO_4_, 1 g of NaCl, 1 g of TSC, and 0.5 g of DHC were added. The mixture was shaken for a further 10 min, then centrifuged at 2500 rcf for 10 min at 21 °C. An aliquot of 6 mL of the supernatant was transferred to a centrifuge tube containing 150 mg of PSA and 2 g of Na_2_SO_4_. The tube was shaken for 10 min and centrifuged again at 2500 rcf for 10 min at 21 °C. A 5 mL portion of the extract was further purified by gel permeation chromatography (GPC; LC-20 Prominence, Shimadzu, Tokyo, Japan) under the following conditions: mobile phase—cyclohexane/ethyl acetate (1:1, *v*/*v*); flow rate—3 mL/min; detection wavelength—254 nm; injection volume—2 mL; fraction collection start time—26 min; and fraction collection end time—47 min. The eluate collected between 26 and 47 min was pooled and evaporated to dryness using a concentrator under a gentle stream of nitrogen (12 ± 2 psi) at 35 ± 5 °C. The residue was dissolved in 500 µL of cyclohexane and transferred to GC vials for analysis.

For multiresidue analysis of LC-amenable pesticides in honey and comb honey, the QuEChERS method was used. A 5 ± 0.1 g portion of the sample was weighed into a 50 mL centrifuge tube and spiked with appropriate amounts of pesticides in working solutions and a mixture of internal standards (amitraz-D12, dichlorprop-D6, carbendazim-D3). Water (5 mL) and acetonitrile (10 mL) were added to the sample, which was then shaken for 10 min. For extraction, Citrate Kit 1 was added, and the sample was shaken again for 10 min, then centrifuged at 2500 rcf for 10 min at 21 °C. For sample clean-up, a 6 mL aliquot of the supernatant was transferred to a 15 mL centrifuge tube containing PSA-Kit-02. The tube was shaken for 10 min and centrifuged at 2500 rcf for 10 min at 21 °C. Prepared extracts were transferred to HPLC vials for analysis.

For the SRM pesticide analysis, the sample was weighed into a 50 mL plastic centrifuge cuvette and processed following the QuPPe method [17]. After spiking with appropriate amounts of polar pesticides in working solution and a mixture of internal standards (glyphosate-13C2,15N, glufosinate-D3, and MPPA-D3), 10 mL of 1% formic acid (*v*/*v*) was added, and the mixture was vigorously shaken by hand or in a rotary shaker for 5 min. After centrifugation at 4000 rpm for 15 min at 4 °C, the aqueous layer was filtered through a regenerated cellulose filter (13 mm, 0.22 μm) into an HPLC vial and subjected to LC-MS analysis.

#### 2.2.3. Quantitative Analysis

The GC–MS/MS system was equipped with an Agilent 8890 gas chromatograph (Palo Alto, CA, USA), a 7693B autosampler, a split/splitless injector in solvent vent mode, and a 7010B tandem mass spectrometry detector with an electron impact ionization source. Chromatographic separation was carried out on two HP-5 MS UI capillary columns (15 m × 0.25 mm ID, 0.25 µm, Agilent Technologies, USA) with helium (99.9999% purity) as the carrier gas at a constant flow rate of 0.8 mL/min. The injection volume was 2 µL. The oven temperature program was: initial temperature 60 °C held for 1 min, ramped at 40 °C/min to 170 °C, then at 10 °C/min to 310 °C. The analysis run time was 20.75 min. Other operating conditions were inlet temperature set to 70 °C for 0.04 min, then ramped to 325 °C at 600 °C/min; transfer line temperature 280 °C; source temperature 300 °C; MS1 and MS2 quadrupole temperatures 150 °C; collision gas (N2) flow 1.5 mL/min; quench gas (He) flow 4.0 mL/min.

Liquid chromatography was carried out using an Agilent Technologies UHPLC 1290 Infinity II and a Triple Quad LC/MS 6460 mass spectrometer equipped with an electrospray ionization (ESI) source (Santa Clara, California, USA). Analytes were separated on an Agilent Zorbax Eclipse Plus C18 column (2.1 × 150 mm, 3.5 μm) maintained at 25 °C. Samples were stored in an autosampler at 10 °C, with an injection volume of 5 μL and a needle wash function. The mobile phase comprised 5 mM ammonium formate (A) and LC-MS grade methanol (B) with the following gradient: 0–5 min, 95% A; 5–25 min, ramp to 50% B; 25–28 min, held at 100% B; 28–28.2 min, return to 95% A. The total chromatographic run time was 33.2 min. Scanning was performed using the multiple reaction monitoring method with at least two transition ions, which, together with retention time and ion ratio, fulfilled the criteria for analyte identification and confirmation. The mass spectrometry scanning parameters in positive and negative ionization modes were gas temperature 260 °C, gas flow 7 L/min, nebulizer 23 psi, sheath gas temperature 375 °C, sheath gas flow 12 L/min, capillary voltage 4000 V (+ and −), and nozzle voltage 500 V (+)/500 V (−). Fragmentor voltages and collision energies were optimized for each analyte.

The method for determining glyphosate, *N*-acetyl-AMPA, glufosinate, and MPPA residues in honey samples was based on liquid chromatography using a porous graphitic carbon column, Hypercarb (100 × 2.1 mm, 5 μm particle size) from Thermo Scientific (Waltham, MA, USA), thermostatted at 40 °C. The mobile phase consisted of 1.2% formic acid in water (A) and 0.5% formic acid in acetonitrile (B), and was pumped at 0.35 mL/min with the following gradient: 0–4.5 min, 100% A; 4.5–6 min, 98% A; 6–7 min, 100% A. Including a 3 min re-equilibration step, the total analysis run time was 10 min. The injection volume was 5 μL. To prevent the analytes from binding to metal surfaces, which can cause false-positive results, peak tailing, and poor recovery, all metal lines in the LC system were replaced with plastic capillaries (such as PEEK). Analytes were introduced into the mass spectrometer using positive ionization mode for glyphosate, glufosinate and MPPA, and negative ionization mode for *N*-acetyl-AMPA. The other MS parameters were gas temperature 275 °C, gas flow 10 L/min, nebuliser 40 psi, sheath gas temperature 400 °C, sheath gas flow 10 L/min, capillary voltage 2000 V (positive and negative), and nozzle voltage 700 V (positive)/1000 V (negative).

All quantitative and qualitative analyses were performed with MassHunter software, based on the two most intense precursor ion–product ion MRM transitions. Procedural standard calibration was used. The analyses were conducted in accordance with all requirements for analyte identification by MS/MS established by SANTE 11312/2021 v2 [18].

#### 2.2.4. Validation Data and Quality Control

The LC-MS/MS and GC-MS/MS methods were validated for specificity, selectivity, repeatability, reproducibility, recovery, and sensitivity in accordance with the SANTE guidance documents applicable at the time of the study [19,20]. Analyte confirmation was based on retention time (RT) and ion ratio criteria. Detailed information on the compounds analyzed is given in Supplementary Table S2. The limit of quantification (LOQ) was defined as the lowest spiking level at which the acceptance criteria specified in SANTE were fulfilled. Repeatability was expressed as the relative standard deviation (RSDr, %) of results obtained from six blank honey samples fortified at a minimum of two concentration levels: one at the LOQ and another at a higher level corresponding to 2–10× LOQ.

These spiked samples were prepared and analyzed on the same day using the same instrument and operators. Within-laboratory reproducibility was expressed as the relative standard deviation (RSDwR, %) and evaluated using two additional sets of blank samples fortified at the same concentration levels as those used for repeatability. These samples were analyzed on two different days using the same instrument but by different operators. Mean recoveries were assessed in the repeatability experiment by comparing the measured concentrations with the fortified concentrations and ranged from 70% to 120%.

Method performance was further verified by participation in an international proficiency test organized by the European Union Reference Laboratory (EURL) for Residues of Pesticides in Food of Animal Origin and Commodities with High Fat Content (CVUA Freiburg, Germany)—EUPT-AO18, and the EURL for Single Residue Methods (CVUA Stuttgart, Germany)—EUPT-SRM18. Quality control during routine analysis was ensured through recovery checks. Blank honey and comb honey samples, previously confirmed to be pesticide-free, were spiked at concentrations corresponding to the LOQ or 2–10 × LOQ within each analytical batch. Recoveries of all analytes were monitored in every batch. In addition, one procedural blank (solvent without matrix) and one blank honey and comb honey sample were analyzed in each batch to confirm the absence of contamination during sample preparation and instrumental analysis.

### 2.3. Trace Elemental Analyses

#### 2.3.1. Chemicals and Standards

All reagents were of analytical grade unless otherwise specified. Double-deionized water produced by a Milli-Q system was used for all dilutions. For instrument calibration, certified standards containing calcium (Ca), Fe, potassium (K), magnesium (Mg), and sodium (Na) at 1000 mg/L each, along with 20 additional trace elements Al, Ag, As, barium (Ba), beryllium (Be), Cd, cobalt (Co), chromium (Cr), Cu, Mn, molybdenum (Mo), Ni, Pb, antimony (Sb), Se, thallium (Tl), vanadium (V), Zn, thorium (Th) and uranium (U) (Environmental Calibration Standard, Agilent Technologies, Santa Clara, CA, USA) at a concentration of 10 mg/L were used. Stock solutions for ICP-MS analysis were prepared by dissolving the multi-element standard mixture in ultrapure water. Working solutions were prepared by serial dilution of the stock solutions with 5% *v*/*v* HNO_3_ (TraceMetal™ Grade, Fisher Chemical, Pittsburgh, PA, USA) and kept at room temperature until use. The certified standard containing bismuth (Bi), indium (In), scandium (Sc), yttrium (Y) and terbium (Tb) (Inorganic Ventures, Blacksburg, VA, USA) at a concentration of 20 mg/L was used as the internal standard. Use of the internal standard is recommended in routine ICP-MS analysis to compensate for possible drift during long-term runs and to correct for matrix effects.

To prepare Hg working standards, 1 mL of concentrated HNO_3_ (65%), 0.1 mL of 10% K_2_Cr_2_O_7_ (Sigma-Aldrich, Steinheim, Germany), and 0.1 mL of concentrated hydrochloric acid (HCl, 37%, Merck, Darmstadt, Germany) were added to each working standard, which was prepared in a brown glass volumetric flask.

#### 2.3.2. Sample Preparation

For microwave digestion, approximately 0.5 g of each sample was accurately weighed and transferred into a pre-cleaned Teflon digestion vial. Then, 1 mL of H_2_O and 2.5 mL of HNO_3_ (65%) were added. All samples were digested using the ULTRAWAVE Single Reaction Chamber (SRC) Microwave Digestion System (MILESTONE, Sorisole, Italy) with the following program: heating to 220 °C over 20 min, held for 10 min at a maximum of 110 bar and 1500 W. The digested sample from each vial was transferred into a clean 50 mL tube and diluted with 5% HNO_3_ prepared with Milli-Q water to a final volume of 50 mL [21].

#### 2.3.3. Quantitative Analysis

The concentrations of Ag, Al, As, Ba, Cd, Co, Cr, Cu, Fe, Mn, Mo, Ni, Pb, Se, V and Zn in the samples were quantified using an inductively coupled plasma instrument with a mass detector, Agilent ICP-MS system Model 7900 (Agilent, Palo Alto, CA, USA). Optimized instrumental conditions for element analysis are presented in Supplementary Table S5. All flasks used in the experiments were drained for 24 h with 5 M HNO_3_ and then thoroughly washed with deionized water. The calibration curve was prepared using at least four points, each measured in triplicate [22].

Total Hg concentration was measured using an in-house method with a solid sample atomic absorption spectrometer, AMA-254 (Advanced Mercury Analyzer, Leco, Katowice, Poland), which does not require chemical pre-treatment. Analyzed sample masses of 0.1 g were placed in the AMA-254 spectrometer in a nickel boat, dried at 120 °C for 70 s, combusted in an oxygen atmosphere at 650 °C for 150 s, and, after a 45 s waiting period (the time required for system cleaning), the next sample was introduced.

#### 2.3.4. Validation Data and Quality Control

As part of the validation process, the parameters include accuracy, precision (repeatability, reproducibility, repeatability of measurement and weighing), limit of detection (LOD), LOQ, and specificity (matrix effects).

Blank samples and certified reference material (Apple leaves, NIST 1515) were included in the same batch as the samples to ensure result quality. As no certified standards for honey are commercially available, the accuracy of the analytical procedures was verified by analyzing certified reference material (CRM) purchased from the National Institute of Standards and Technology (NIST).

### 2.4. Statistical Analysis

Statistical analyses were performed in Python, version 3.12 (Python Software Foundation, Wilmington, DE, USA), using Visual Studio Code, version 1.108.0 (Microsoft Corporation, Redmond, WA, USA), and in R (R Foundation for Statistical Computing, Vienna, Austria) using RStudio (2025.09.1+401 and R version 4.5.1.) (Posit PBC, Boston, MA, USA).

For a detailed analysis of pesticides, boxplot visualization was used to examine compound-specific differences between comb honey and honey by analyzing paired samples from the same locations. As residue levels can vary greatly and often differ by several folds rather than by small absolute amounts, a logarithmic transformation was used instead of simply subtracting concentrations. This approach allows clearer comparison of proportional differences and reduces the influence of very large values. The observed differences were further tested using paired Wilcoxon tests and Spearman correlations of pesticide concentrations within and between matrices for each pesticide at the site level.

For the statistical analysis of metal concentrations in honey and comb honey samples, two methods were used: Principal Component Analysis (PCA) and Spearman correlation analysis. PCA summarized the overall variability in the dataset by reducing numerous metal variables to a smaller number of components, enabling identification of patterns and similarities between samples, as well as the metals driving these patterns. Before PCA, the data were log-transformed and standardized. Spearman correlations were also calculated between metals to assess pairwise relationships. Spearman correlation was selected because it is rank-based and more robust for non-normally distributed data and potential outliers, which are common in concentration datasets.

Given the characteristics of the dataset, which posed challenges for the application of formal censored-data methods, censored values were handled using a simple imputation approach, with missing values (<LOQ) replaced by the median to enable principal component analysis (PCA). Correlation analyses were conducted using pairwise complete observations and comparative analyses were based on available paired data. Therefore, this part of the analysis should mainly be viewed as exploratory and not as robust statistical tests.

## 3. Results and Discussion

### 3.1. Pesticide Analysis

A total of 22 honey and 38 comb honey samples were analyzed for 190 pesticide residues from various chemical classes with different modes of action, demonstrating a comprehensive approach to assessing the presence of these substances in honey. The achieved sample size (n = 60 honey samples from 20 apiaries) ensured coverage of the main exposure hotspots in the region and is comparable to or higher than sample sizes used in previous studies investigating pesticide residues in honey [4,8,13]. The methodology enabled assessment of whether the results complied with the established MRLs for honey and other apiculture products [23], which are based on experimental residue trials and dietary risk assessment data. It is important to note that these MRLs do not apply to other apiculture products, such as comb honey, as the consumption and daily intake of this in-hive material are considered negligible compared to honey, which limits the risk to human health [24].

The results showed that all honey samples contained at least one pesticide residue at concentrations above the LOQ (Table S6). The largest proportions of samples contained residues of three pesticides (36.4%) and four pesticides (27.3%). In total, six pesticide active substances and metabolites were identified: acetamiprid, coumaphos, DMF, thiacloprid, glyphosate, and DMPF, with concentrations ranging from 0.001 mg/kg to 0.076 mg/kg. Although acetamiprid is the only neonicotinoid approved for use in open agricultural areas in the EU [25], it was not the only detected member of this group, as thiacloprid was found in one honey sample from Međimurje County. The observed concentration was slightly above the LOQ, but its detection, despite being banned since 2020 [26], may indicate its stability and persistence in the environment or possible unauthorized agricultural use. Glyphosate was the only substance detected in three honey samples at concentrations above the MRL (0.05 mg/kg) in this study. It was found exclusively in samples from Varaždin County, and its presence often reflects widespread environmental use through herbicide spraying in agricultural or public areas.

Analysis of comb honey samples identified 21 pesticides in total, with a detailed list and concentration ranges summarized in Table S7. To assess regulatory relevance, the concentrations in comb honey were compared with established MRL thresholds for honey. Fipronil-sulfone exhibited the most pronounced pattern, with all 19 comb honey samples exceeding the reference value of 0.005 mg/kg. Residues of this compound in comb honey were up to 12 times higher than the MRL for honey, while no detections were observed in the corresponding honey samples, indicating a systematic reduction after extraction. The reason for this “fortunate” circumstance, and the lack of transfer to extracted honey, which would have resulted in much greater consumer exposure to this persistent and even more toxic metabolite than fipronil itself [27], is that this compound is highly lipophilic and tends to bioaccumulate in lipid-rich hive matrices, particularly in beeswax. Coumaphos as the active ingredient of registered veterinary medicines, is an insecticide widely used in beekeeping to control Varroa destructor mite infestations, ranged from 0.011 mg/kg to 1900 mg/kg in comb honey, with over 50 percent of results above 0.1 mg/kg (MRL for honey). Similarly, the prevalence of amitraz in honey and comb honey is expected due to the intensive use of this compound as a veterinary medicinal product (VMP) but also as unapproved homemade formulations for inappropriate administration. The current residue definition for amitraz includes metabolites containing the 2,4-dimethylaniline moiety, mainly DMF and DMPF, which are almost exclusively present in honey and other honey bee products because amitraz degrades rapidly within 15 days of application [28]. While the highest concentrations of metabolites in honey were around 0.03 mg/kg, one comb honey sample contained an elevated concentration of DMPF at 255.8 mg/kg. An interesting case involved a sample 16 COMB HONEY 36 from Varaždin County, which despite having concentrations of amitraz metabolites below the MRL for honey of 0.2 mg/kg (DMF—0.030 mg/kg; DMPF—0.132 mg/kg), would still exceed this limit because the sum of all concentrations contributes to the total, calculated using the molar mass ratio. An unexpected finding from the analysis of comb honey samples was the presence of the fungicide trifloxystrobin in all samples, with concentrations ranging from 8.8 mg/kg to 90.9 mg/kg. In contrast, trifloxystrobin was not detected in honey samples above the LOQ, likely due to its lipophilic properties (log P 4.5), which cause it to partition into and accumulate in beeswax during honey extraction [29].

**Figure 2:**
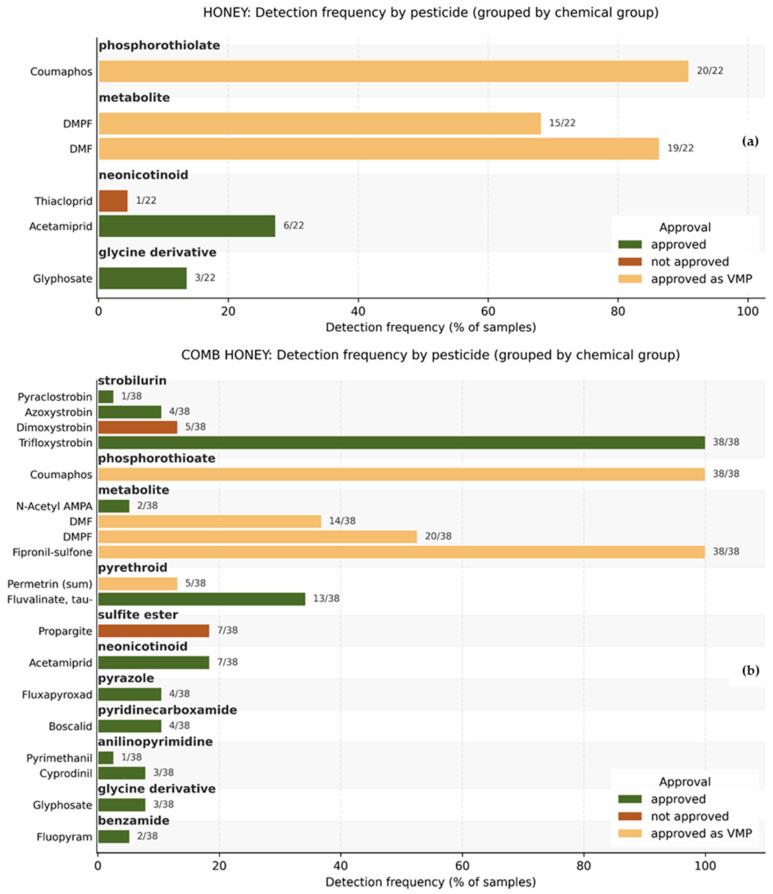
Detection frequency of pesticides in honey (**a**) and comb honey (**b**) samples.

Most of the detected pesticides are authorized for use in the EU, either as PPPs or VMPs, although banned active substances such as dimoxystrobin and propargite (previously used as an acaricide) were also present in our study. Figure 2 shows the detection frequency of each pesticide (percentage of samples above the LOQ) in honey and comb honey samples, their chemical group, and EU regulatory approval status (color-coded).

**Figure 3:**
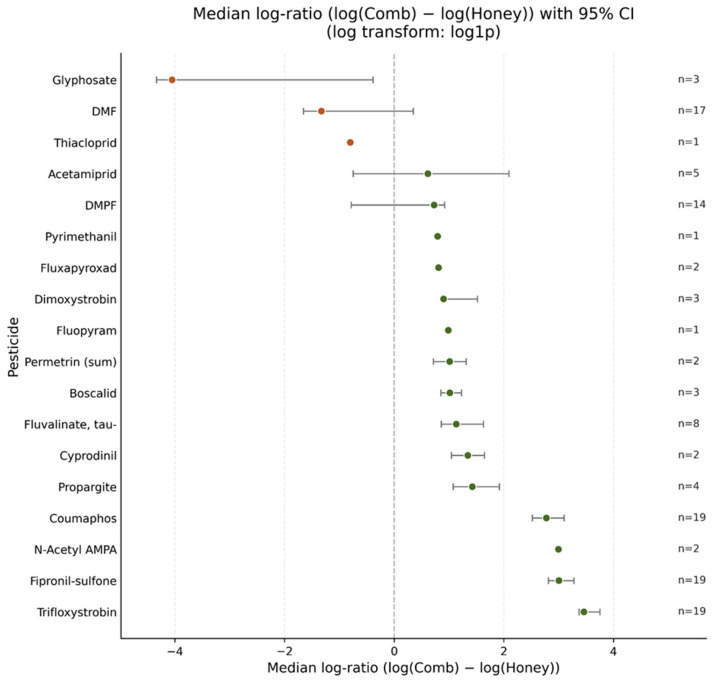
Median paired log-differences in pesticide concentrations between honey and comb honey across locations using paired Wilcoxon signed-rank tests.

To compare pesticide residues between comb honey and extracted honey in greater detail, we analyzed paired samples from the same locations. Due to the unbalanced sampling design, all comparisons between honey and comb honey were carried out at the level of sampling locations. Only locations with data available in both matrices were included in the paired analyses, resulting in variations in the number of paired observations between pesticide compounds. The forest plot in Figure 3 summarizes the median log-ratio across locations for each pesticide, together with 95% bootstrap confidence intervals. Several compounds show clearly positive effects (shifted above zero), indicating consistently higher concentrations in comb honey compared to honey. The strongest and most consistent patterns were observed for trifloxystrobin, fipronil-sulfone, and coumaphos. For these pesticides, confidence intervals were clearly separated from zero, suggesting a systematic matrix-dependent distribution pattern. The vast majority of detected substances (such as acetamiprid, DMPF, boscalid, and propargite) had estimates closer to zero or wide confidence intervals, indicating either smaller effects or greater variability across locations. For some pesticides with very few paired locations, estimates should be interpreted cautiously due to limited statistical power. DMF and glyphosate were shifted below zero, indicating higher concentrations in honey than in comb honey. However, for DMF, the confidence interval included zero, indicating no statistically robust difference. For glyphosate, the available paired data indicated higher concentrations in extracted honey than in comb honey, but this pattern was based on only three paired samples and should therefore be regarded as exploratory and interpreted with great caution.

To formally test whether the observed differences were statistically consistent across locations, we applied paired Wilcoxon signed-rank tests to the location-level log-ratios. This non-parametric approach does not assume a normal distribution of differences and is therefore appropriate for skewed environmental concentration data. To account for multiple testing across pesticides, *p*-values were adjusted using the Benjamini–Hochberg false discovery rate (FDR) procedure [30]. Pesticides that remained statistically significant after FDR correction (q < 0.05) are shown in Table 1. The combination of graphical and inferential analyses demonstrates that trifloxystrobin, fipronil-sulfone, coumaphos, and fluvalinate (tau-) display robust and systematic enrichment in comb honey compared with honey, whereas other compounds exhibit weaker or inconsistent differences.

**Figure 4:**
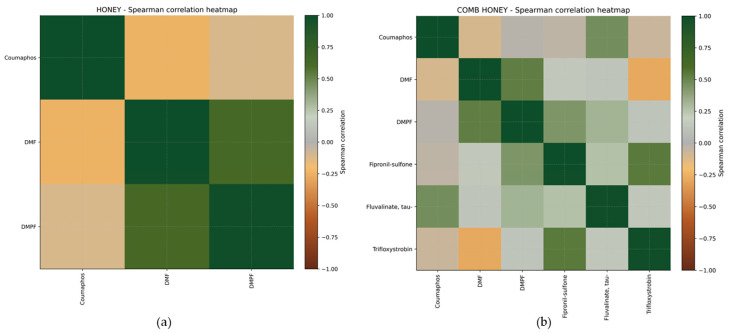
Spearman correlation heatmap of pesticide concentrations in honey (**a**) and comb honey (**b**) samples, where darker red indicates stronger positive correlations and darker green indicates stronger negative correlations between compounds.

To assess whether certain compounds tend to occur together across sampling sites, we analyzed Spearman correlations between pesticide concentrations within each matrix. In honey (Figure 4a), only the correlation between DMPF and DMF remained statistically significant after FDR correction, again reflecting their metabolic linkage, while other associations were weak and not statistically supported, indicating less pronounced clustering of compounds within the honey matrix. In comb honey (Figure 4b), several moderate positive correlations were observed between pesticide pairs, although none remained significant after FDR correction. The strongest association was between fipronil-sulfone and trifloxystrobin (ρ = 0.54), followed by the correlation between the amitraz metabolites DMF and DMPF (ρ = 0.53), and between coumaphos and tau-fluvalinate (ρ = 0.46). Weak negative correlations were also detected (e.g., DMF vs. trifloxystrobin, ρ = −0.29), indicating that higher levels of one compound tend to occur in samples where the other is lower or absent.

**Figure 5:**
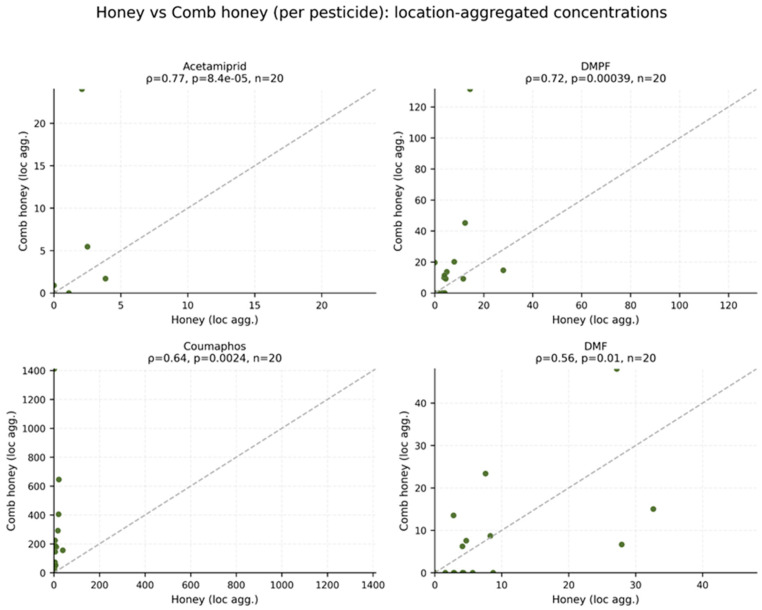
Scatter plots showing the relationship between comb honey and honey concentrations (aggregated per location) for acetamiprid, coumaphos, DMPF and DMF.

Spearman correlations were also calculated between matrices for each pesticide at the location level. In Figure 5, each panel displays the Spearman correlation coefficient (ρ) and *p*-value, with the dashed line indicating the 1:1 reference line to illustrate deviations between matrices. Several compounds showed strong and statistically significant positive correlations between matrices. The highest association was observed for acetamiprid (ρ = 0.77), followed by DMPF (ρ = 0.72), coumaphos (ρ = 0.64), and DMF (ρ = 0.56), all of which remained significant after FDR correction. These results indicate that, despite differences in absolute concentration levels between matrices, relative spatial patterns were partly preserved: locations with higher residue levels in comb honey also tended to have higher levels in honey.

### 3.2. Trace Element Analysis

Collected honey and comb honey samples were tested for 17 trace elements. The concentrations in comb honey samples tended to be higher than those in honey, confirming that wax, owing to its lipid-based absorbent structure, serves as a long-term sink for heavy metals. Elements such as Cu, Fe, Mn, Se, and Zn in recommended amounts, may have antioxidative properties and a generally beneficial effect on human health. In contrast, As, Cd, Cr, and Pb, among others, have no known benefits and can be toxic even in relatively small quantities [31]. According to Commission Regulation (EU) 2023/915, honey as a food product must meet only the maximum level requirement for Pb (0.10 mg/kg), while no limit has been established for other elements [32].

In this study, one honey sample and 14 comb honey samples contained Pb at quantifiable levels, with a maximum concentration of 0.088 mg/kg, confirming compliance with legislation. Copper, Ni, and Zn were detected in all analyzed samples, while As, Ag, and Se levels were not detected in any sample. Vanadium and Co showed a similar pattern; each exhibited only one quantified value in honey, but both were detected above the LOQs in three of four comb honey samples from the same locations. The decreasing order of concentrations in honey (Fe > Al > Zn > Ni > Mn) changed in comb honey due to a noticeable increase in Mn levels (from 0.28 mg/kg to 2.05 mg/kg). Manganese was detected in all honey samples, with a maximum value of 0.79 mg/kg, and 76% of the samples contained less than the mean value of 0.28 mg/kg. In comb honey, Mn was present at higher concentrations, ranging from 0.194 to 14.9 mg/kg. Manganese is a natural component of honey, and its content plays a crucial role in identifying honey varieties [11], but it can also be influenced by the bees themselves [33]. Iron was the most abundant metal analyzed in the study. Although, the Fe content ranged from 0.198 mg/kg to 17.2 mg/kg in comb honey samples, with an average concentration of 5.92 mg/kg, the highest Fe concentration was found in the honey sample from location 7 in Međimurje County. As most of the selected sampling locations are not near roads with heavy vehicular traffic or industrial plants, it is assumed that the main source of Fe content in honey is the environment, through contaminated air and soil. In areas with acidic soil or industrial pollution, bees can be exposed to Al during foraging, and honeybee products may also be contaminated by equipment such as Al containers or extractors used in honey processing. In this study, the overall mean Al concentration was 3.24 mg/kg, with maximum values of 2.07 mg/kg in honey samples and 21.4 mg/kg in comb honey samples. Chromium and Ba, detected in over 70% of all analyzed samples, showed narrower concentration ranges below 1 mg/kg in both matrices. Chromium levels were higher in honey samples, averaging 0.159 mg/kg, compared with 0.077 mg/kg in comb honey, while Ba concentrations ranged from 0.012 mg/kg to 0.179 mg/kg in honey and from 0.023 mg/kg to 0.714 mg/kg in comb honey. Zinc levels generally vary depending on the floral source and region, with darker honeys such as chestnut or honeydew honey containing higher mineral content than lighter, sweeter varieties [13]. The mean Zn content in honey across all 22 samples analyzed in this study was 0.61 mg/kg, with the highest value recorded at 1.36 mg/kg. In comb honey, Zn concentrations varied considerably, ranging from 0.266 mg/kg to 33.0 mg/kg. In some cases, this contribution does not originate solely from agricultural and environmental sources, but may result from the transfer of metals to the honey via old honeybee frames or contaminated wax (slumgum) [34]. Although analysis confirmed the presence of Cu in all samples, the observed concentrations were low, with the highest values being 2.98 mg/kg in comb honey and 0.295 mg/kg in honey, indicating no contamination during storage or processing, such as from metal storage equipment. Nickel levels in honey may indicate that bees forage on plants which accumulate Ni from the soil. As this study found similar mean concentrations of this element in both honey (0.32 mg/kg) and comb honey (0.27 mg/kg) samples, these results may indicate a lack of significant industrial pollution in the sampled area.

Regarding the origin of the detected metal(oid)s, pesticide formulations may have also contributed in some cases. In addition to the active substance, formulations can contain other ingredients or impurities, and some crop protection products are inherently metal-based, particularly Cu fungicides [35]. Furthermore, metal contaminants such as As, Co, Cr, Ni, and Pb have been found in commercial pesticide formulations [36]. In our study, the elevated concentrations of glyphosate in three honey samples from Varaždin County could not be clearly linked nor could it be concluded that the concentration of any metal in the honey samples from those locations was influenced by the use of this PPP.

The results of the univariate analysis of the element concentrations in the honey and comb honey samples (LOQ, minimum and maximum values, median, mean, standard deviation) are presented in Table 2.

**Figure 6:**
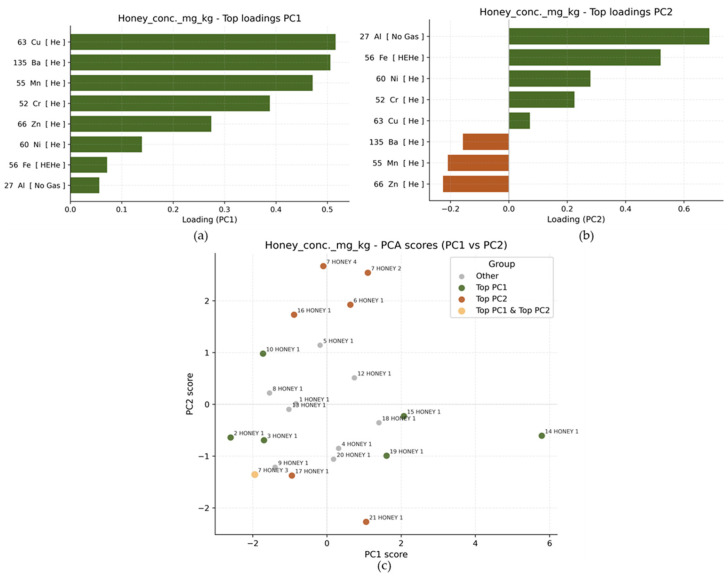
Loadings of metals on the PC1 (**a**) and PC2 (**b**) for the honey dataset; PCA score plot (PC1 vs. PC2) for honey samples (**c**).

To further simplify the metal datasets through dimensionality reduction, PCA was conducted. The first two principal components accounted for most of the information (PC1: 39.7% and PC2: 21.3%), indicating that a large part of the variation between samples can be described in just two dimensions. PC1 primarily reflects variation driven by Cu, Ba, Mn, and Cr, which have the strongest positive loadings (Figure 6a). This shows that samples with high PC1 scores tend to have relatively higher concentrations of these metals compared to other samples. PC2 separates samples mainly based on differences in Al and Fe and Ni, which have the highest loadings in this component (Figure 6b).

In the PCA score plot (Figure 6c), the most extreme honey samples, with the highest and lowest absolute scores for PC1 and PC2, were highlighted to represent those that differ most from the overall average composition. Notably, these samples do not form separate clusters but appear as individual outliers within an otherwise homogeneous dataset. For PC1, the highest scores are found in 19 HONEY 1, 15 HONEY 1, and 14 HONEY 1, mainly due to elevated concentrations of Cu, Mn, Ba, and Cr. Conversely, samples 2 HONEY 1 and 3 HONEY 1 have strongly negative PC1 scores, indicating lower relative levels of these elements. For PC2, samples 7 HONEY 4, 7 HONEY 2, 6 HONEY 1, and 16 HONEY 1 have high scores, suggesting relatively higher Al and Fe concentrations compared to the rest. In contrast, lower levels of Al and Fe in samples 21 HONEY 1 and 17 HONEY 1 result in low PC2 scores. Interestingly, 7 HONEY 3 has low values for both PC1 and PC2, indicating generally lower concentrations of the metals influencing both components. Overall, the highlighted samples help identify which specific honeys deviate most from the typical metal profile. However, the absence of clearly separated clusters indicates that variation is driven by individual extreme samples rather than by distinct subgroups. This suggests local differences rather than systematic groupings within the honey dataset.

**Figure 7:**
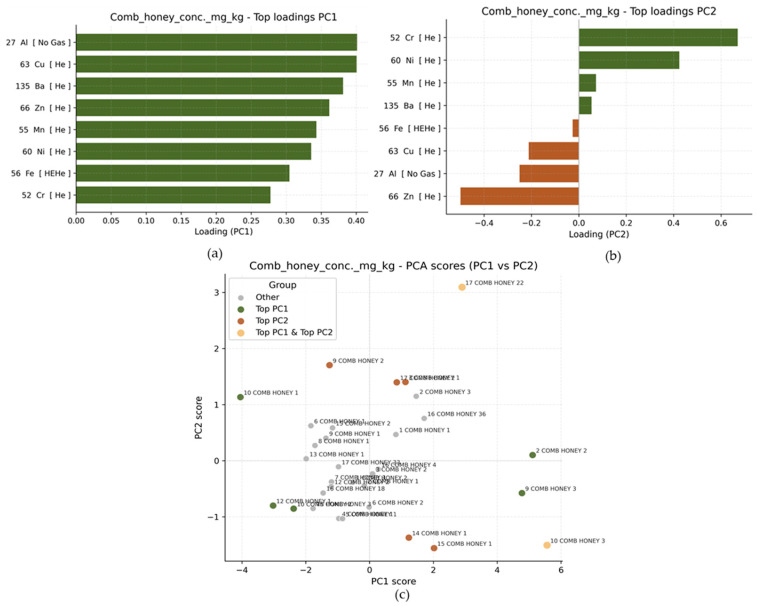
Loadings of metals on the PC1 (**a**) and PC2 (**b**) for the comb honey dataset; PCA score plot (PC1 vs. PC2) for comb honey samples (**c**).

The principal components in the comb honey dataset explained most of the total variance, with PC1 accounting for 62.1% and PC2 for 13.2%. This shows that an even larger proportion of the differences between samples is captured primarily along a single dominant axis (PC1). In other words, most of the variability in comb honey metal composition is driven by one main pattern. PC1 mainly reflects variation associated with Al, Cu, Ba, Zn, Mn, Ni, and Fe, all of which have relatively strong positive loadings (Figure 7a). Thus, PC1 in the comb honey dataset represents a general “overall metal concentration” gradient, where samples with high PC1 scores tend to have higher concentrations of most measured metals simultaneously. Conversely, samples with low PC1 scores generally show lower metal concentrations across multiple elements. PC2 is mainly influenced by Cr and Ni, which have the strongest positive loadings, while Zn and Al contribute negatively (Figure 7b). Therefore, PC2 separates samples primarily by contrasting higher Cr and Ni levels with higher Zn and Al levels.

In the PCA score plot (Figure 7c), samples 17 COMB HONEY 22 and 10 COMB HONEY 3 differ significantly from the average metal composition along both main axes of variation and are therefore highlighted in both directions. For PC1, the highest values are observed for 2 COMB HONEY 2 and 9 COMB HONEY 3, indicating that these samples have generally elevated concentrations of the metals driving PC1 (Al, Cu, Ba, Zn, Mn, and Ni), while samples 10 COMB HONEY 1, 12 COMB HONEY 1, and 10 COMB HONEY 2 have negative PC1 values, indicating comparatively lower overall metal concentrations. For PC2, higher values are observed for 9 COMB HONEY 2, 17 COMB HONEY 2, and 3 COMB HONEY 1, suggesting relatively higher Cr and Ni levels compared to Zn and Al. Lower PC2 values are found in 14 COMB HONEY 1 and 15 COMB HONEY 1, indicating the opposite trend, with relatively lower Cr and Ni and higher Zn and Al influence. As with honey, the highlighted samples again represent individual outliers rather than distinct clusters, indicating that variability in comb honey is driven by specific extreme samples rather than by clearly separated groups.

**Figure 8:**
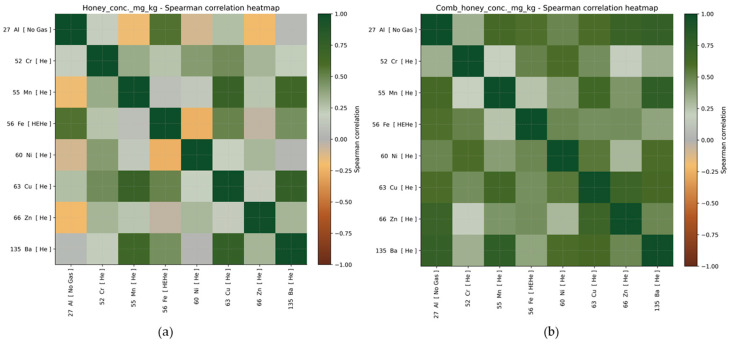
Spearman correlation heatmap of metal concentrations in honey (**a**) and comb honey (**b**) samples, where darker red indicates stronger positive correlations between metals, and darker green indicates stronger negative correlations.

The Spearman correlation analysis (Figure 8a) in honey reveals several moderate to strong positive relationships between metals. The strongest correlations are between Cu and Ba (r ≈ 0.75) and Cu and Mn (r ≈ 0.73), indicating that these metals tend to increase together across samples. Manganese is also strongly correlated with Ba (r ≈ 0.67), while Fe shows a moderate positive correlation with Al (r ≈ 0.57). These findings are consistent with the PCA results. The metals that are strongly correlated (Cu, Mn, Ba, and Cr) are the same metals that primarily drive PC1, supporting the interpretation that PC1 represents a joint enrichment pattern of these elements. Similarly, the positive association between Al and Fe supports the structure captured by PC2, which is mainly influenced by these two metals.

The Spearman correlation analysis in comb honey (Figure 8b) shows several strong positive relationships, such as between Mn and Ba (r ≈ 0.78), Al and Ba (r ≈ 0.75), and Al and Zn (r ≈ 0.72). Additionally, Cu is strongly correlated with Zn (r ≈ 0.69) and Mn (r ≈ 0.65), while Ni shows moderate to strong positive correlations with Ba (r ≈ 0.59) and Cr (r ≈ 0.59). Most correlations are positive, indicating a common enrichment pattern across multiple metals rather than opposing trends. These findings are also consistent with the PCA results. The metals showing strong mutual correlations (Al, Mn, Ba, Cu, and Zn) are the same metals that primarily drive PC1, supporting the interpretation that PC1 represents a general metal enrichment gradient. Furthermore, the moderate positive association between Cr and Ni aligns with their importance in PC2.

### 3.3. Comparison with Pesticide and Trace Element Concentrations in Honey from Other Studies

Numerous papers address the issue of extensive pesticide use and its detrimental effects on honeybee health, as well as the residues of these compounds that remain in honeybee products and pose a potential risk of consumer exposure to environmental toxins [5]. The results of such research can be particularly interesting and provide answers to many questions when the material analyzed originates from hotspot areas, as in this study. By reviewing the results across pesticide classes, we can highlight the frequent co-occurrence of insecticides (acetamiprid, thiacloprid) and fungicides (trifloxystrobin, boscalid) with the most prominent group, the acaricides (amitraz, coumaphos), mostly present due to apiary management. Combinations of detected active substances are often influenced by agricultural and apicultural practices, where PPPs frequently overlap with VMPs. This was clearly demonstrated in the study by Murcia Morales et al. [37], which detected 31 chemical residues in the in-hive samples, although only amitraz was applied to the honey bee colonies. Strong associations between acaricides, mainly DMF-DMPF-coumaphos, observed in our study can be easily explained by their high popularity and widespread use in beekeeping worldwide. Another study from Croatia also quantified levels of amitraz (as the sum of DMF and DMPF metabolites) in approximately one third of conventional honey samples and in one organic honey sample, with concentrations ranging from 0.005 mg/kg to a maximum of 0.037 mg/kg [38]. Coumaphos was detected even more frequently, in 80% of conventional honey and 12% of organic honey samples, although the latter should be completely free of coumaphos. None of the samples contained residues at levels above the EU MRLs, as was also found in Israeli [8], Slovenian [39] and Spanish [40] honey. Interestingly, in their analysis of 223 pesticides in 30 samples of varietal honey from south-eastern Poland, Kędzierska-Matysek et al. [13] did not detect any coumaphos residues. The neonicotinoid group predominated, with acetamiprid and thiacloprid present in almost 90% of the samples. The mean concentrations of these compounds were 0.013 mg/kg and 0.053 mg/kg, respectively, which are somewhat higher than the concentrations found in our samples from northern Croatia (0.003 mg/kg and 0.001 mg/kg, respectively). In a study from Greece [10], 19 active substances were detected in total. Although several pyrethroids, such as cypermethrin and cyfluthrin, were present, the highest concentrations were found for imidacloprid, an insecticide still approved for use during the sampling period, which exceeded the MRL (0.05 mg/kg) in two cases (0.29 mg/kg and 0.78 mg/kg). We observed exceedances of the MRL only for glyphosate in three honey samples from Varaždin County, indicating its agricultural and/or domestic use in that specific region. As concentrations ranged from 0.056 mg/kg to 0.076 mg/kg, the samples are still considered compliant due to the uncertainty calculation in accordance with the SANTE guidance document [18]. Three-year monitoring results for honey from the Lombardy and Emilia-Romagna regions in Italy [41] showed the presence of this herbicide in approximately 28% of analyzed samples, with several values above the MRL; however, only two samples (0.25 mg/kg and 0.31 mg/kg) were definitively declared unsafe for human consumption. Rampazzo et al. [42,43] monitored glyphosate, glufosinate, and their metabolites in honey from the Italian market. Although a significant percentage of samples contained quantifiable levels of glyphosate, only one sample in each study exceeded the MRL by more than twice (0.14 mg/kg and 0.12 mg/kg, respectively).

Comb honey is a valuable material for assessing the presence and accumulation of pesticides in the hive with which bees come into contact; however, there is limited literature on monitoring this type of sample, mainly due to the lack of specific, harmonized regulations for this product. An investigation of 261 different pesticide residues in natural comb honey from Tokat province in Turkey detected only two active substances in a single sample [4]. Pirimicarb and tebuconazole were found at elevated levels of 0.469 mg/kg and 0.025 mg/kg, respectively, both exceeding the MRL for honey, while all other samples were free of pesticides. These findings are surprising because more than half of the samples were taken from areas near pesticide-use zones, yet even low levels of acaricides, which are commonly present due to beekeeping practices, were entirely absent. A much more similar set of comb honey analysis results to ours was obtained by Ostiguy and Eitzer [44] in their study of samples from various regions of North America. In addition to detecting the insecticides and acaricides coumaphos, tau-fluvalinate, and dimethoate, the presence of the fungicide boscalid was also observed, along with two herbicides, atrazine and bentazone, which were not included in our research. While almost identical levels were observed for boscalid concentrations below 0.005 mg/kg and tau-fluvalinate below 0.030 mg/kg, the concentration of the most abundant coumaphos in our case was nearly seven times higher (1.90 mg/kg compared to 0.280 mg/kg). The concentration levels and, in particular, the detection frequency recorded for trifloxystrobin and fipronil-sulfone in our comb honey samples cannot be compared with other studies, as such data are not available in the literature. While trifloxystrobin, a widely used fungicide in agriculture, generally has low acute toxicity to adult honey bees and is unlikely to pose a risk to consumer health through honey consumption, fipronil sulfone is a persistent oxidative metabolite of the insecticide fipronil and is banned as a seed treatment for most crops in the EU [45]. Fipronil sulfone is as toxic as, or even more toxic to vertebrates [27] than the parent compound, suggesting that its high detection rate in raw honey could be of concern.

To assess honey quality more accurately and representatively, many authors monitor more than one group of pollutants in their research, including pesticides, metals, and other pharmacologically active substances. As trace element levels vary considerably between honeys of different botanical origins, it is necessary to exercise great care when interpreting the results and to ensure that comparisons are made between honeys from the same or similar plant species [46]. This provides a clearer understanding of the levels of trace elements originating from the environment or potentially from anthropogenic sources. Lazarus et al. [38] quantified trace metals and metalloids in nearly all honey samples, with none exceeding the MRL for Pb of 0.1 mg/kg. Only Cr showed a statistically significant difference between organic and conventional honey (*p* = 0.006); the reason for its higher content in organic samples remains unknown. Analysis of Polish varietal honey samples showed that all five heavy metals, Cd, Hg, Pb, Cu, and Zn, were present in 90% of samples, although only at trace levels [13]. It was noted that the honey variety did not affect Zn levels (0.66–2.69 mg/kg), while Cu content was significantly influenced by botanical origin, ranging from 0.50 mg/kg in rapeseed honey to 1.20 mg/kg in buckwheat honey. Concentrations of these elements were several times lower in our study and were much more comparable with the values reported by Šerevičienė et al. [47]. Honey samples collected from urban and potentially contaminated sites generally had low heavy metal content, except for one sample with a Pb concentration above the EU regulatory limit. Although geographically very distant and differing completely in plant species at each location, it is interesting to note the trend in the concentrations obtained for certain elements in our study compared with those from North Africa [48] and South Asia [49]. In all cases, Fe was the most abundant heavy metal, with values of almost 27 mg/kg in Algerian honey, 2.60 mg/kg in Croatian honey, and 1.81 mg/kg in Indian honey. Similarly, the concentrations of other trace elements such as Zn, Mn, and Cu were comparable to those reported by Verma et al. [49] or several times lower than those reported by Bereksi-Reguig et al. [48]. Testing honey from various floral origins at apiaries in southern Italy revealed the presence of all target metals, metalloids, and microelements [11]. Aluminum was the most abundant microelement, with a mean mass concentration of 5.13 mg/kg. Among the toxic metals, Pb had the highest levels (0.11 mg/kg), exceeding the MRL in most analyzed samples. Similar levels of Pb were reported in a previous study from Croatia [50], with all mean concentrations above 0.1 mg/kg. The highest value, 0.40 mg/kg, was found in comb honey from Varaždin, the same region included in this study. Although the authors attributed the high Pb concentration to soil in Varaždin County as a possible source of contamination, we found a maximum value of 0.034 mg/kg in comb honey from that region, suggesting that anthropogenic activities are a more likely cause of these results. Higher levels of Cr in honey compared to comb honey samples were observed in both studies, while for most other trace elements, we found higher content in comb honey; however, this was generally not the case in the 2024 study.

### 3.4. Study Limitations and Future Directions

This study has several limitations that should be considered when interpreting the results. First, sampling was conducted at a single time point (June, after the acacia flow), which does not permit conclusions about intra-annual or inter-annual variability in pesticide and metal concentrations in honey and comb honey. As pesticide application patterns, flowering dynamics, and climatic conditions change throughout the season and between years, the measured levels reflect only a brief, specific exposure window rather than the complete range of possible contamination scenarios.

Second, the study covered a limited number of apiaries situated in an agricultural hotspot with recurrent bee poisoning incidents, and beekeeper participation was voluntary. This targeted design is suitable for identifying potential worst-case or high-risk situations, but it may restrict the generalizability of the findings to all beekeeping operations and honey types in the wider region.

Third, although differences between extracted honey and comb honey were demonstrated, particularly for lipophilic pesticides which showed enrichment in comb honey, the study did not directly investigate the mechanisms of transfer, accumulation, or degradation of these compounds within hive matrices. Similarly, while the presence of glyphosate and neonicotinoids was confirmed, the sources and pathways of contamination were not specifically addressed. For some pesticide active substances and metabolites, the number of paired samples was small, limiting the ability to detect subtle differences between matrices (comb vs. extracted honey) and to perform robust statistical comparisons. The observed patterns for these compounds should therefore be considered exploratory and interpreted with caution.

Fourth, the assessment of food safety in this study was limited to comparing measured concentrations with existing maximum residue limits (MRLs) and toxicological reference values for individual substances. Current regulatory frameworks and risk assessment approaches remain largely focused on single-compound exposures, although consumers are typically exposed to mixtures of multiple pesticides and metals at low concentrations. The potential for additive, synergistic, or antagonistic effects between residues detected in honey and comb honey is not well characterized and could not be addressed within the scope of this study.

Despite these limitations, the data generated provide a valuable baseline for characterizing consumer exposure to pesticide residues and metals through honey and comb honey produced in high-risk agricultural landscapes. Future studies should extend monitoring across multiple seasons and production years, include more apiaries, and apply mixture toxicity and cumulative risk assessment approaches. Such integrated datasets would improve our ability to interpret low-level residues in terms of public health relevance, support refinement of MRLs and guidance values for honey, and ultimately contribute to evidence-based measures for consumer protection and sustainable use of honeybee health and plant protection products.

## 4. Conclusions

This study documented the presence of various pesticides and trace elements in honey samples from northern Croatia, a region that has experienced significant local losses of honey bee colonies and suspected acute poisoning due to pesticide use in recent years. To further investigate honeybee’s exposure and the potential transfer of these pollutants to honeybee products, samples of extracted honey, widely used as a functional food, and comb honey, often served as a delicacy for human consumers, were collected and analyzed.

The analysis of complete spectra of pesticides from different chemical classes in honey samples confirmed the presence of the most prominent and expected acaricides residues (amitraz metabolites DMF and DMPF, and coumaphos) in honey bee products, owing to the intensive use of these substances in beekeeping management. In addition, two neonicotinoids were detected at concentrations slightly above the LOQ, and the herbicide glyphosate was found in three honey samples at levels that may indicate notable contamination, which could raise some concerns regarding consumption. By comparing pesticide concentrations in extracted honey and comb honey, compound-specific differences between the two matrices were observed. For several pesticides, concentrations in comb honey tended to be higher than in extracted honey, suggesting that residues may be retained or enriched in comb honey, whereas concentrations in the extracted honey matrix appear lower, possibly due to dilution (homogenization) or faster removal during extraction and handling. Statistical analysis confirmed that this pattern was particularly evident for trifloxystrobin, fipronil-sulfone, tau-fluvalinate, and coumaphos, which showed consistent and systematic enrichment across locations. In contrast, for compounds such as acetamiprid and DMF, distributions in comb honey and honey largely overlapped, indicating no consistent matrix-related difference within the variability of our dataset.

The residual concentrations of trace elements in honey generally vary significantly depending on regional environmental conditions, floral sources, and proximity to pollution sources. In our study, Fe, Al, Zn, Mn, Cu, and Ba were quantified in almost all samples analyzed and were present at the highest concentrations in both types of honey. Although some studies show that Pb levels in honey exposed to local environmental pressures may exceed permissible limits (0.1 mg/kg), this was not observed in our analyses. Only one honey sample had a concentration of 0.019 mg/kg, and the highest concentration found in comb honey was 0.088 mg/kg. PCA did not reveal systematic variations within the metal dataset; instead, it showed localized differences caused by individual extreme samples. The strongest correlations between metals that tend to increase together across honey and comb honey samples were confirmed for Cu–Ba and Mn–Ba, respectively.

Although this study focused on occurrence and distribution patterns and did not assess the potential combined effects of pesticides and trace elements on human consumption, we consider our sample representative for ecological assessment of high-risk apiaries within the investigated hotspot area. However, we explicitly refrain from extrapolating the results to all apiaries in Croatia or to all honey production seasons.

## Figures and Tables

**Table 1:** Pesticides with statistically significant transfer effects (FDR-adjusted q < 0.05).

Pesticide	n Paired Locations	Median Log-Ratio	95% CI (lo, hi)	Wilcoxon *p*	FDR q
Trifloxystrobin	19	3.46	3.37, 3.75	1.91 × 10^−6^	1.59 × 10^−5^
Fipronil-sulfone	19	3.00	2.81, 3.28	1.91 × 10^−6^	1.59 × 10^−5^
Coumaphos	19	2.78	2.52, 3.10	<0.001	<0.01
Fluvalinate (tau-)	8	1.13	0.86, 1.63	0.0059	0.024

**Table 2:** Element levels in honey and comb honey samples from northern Croatia.

Honey (mg/kg)						
**Element**	**LOQ**	**Min**	**Max**	**Median**	**Mean**	**Std Dev**
**Al**	0.025	0.531	2.060	1.044	1.068	0.428
V *	0.027	0.275	0.275	0.275	0.275	-
**Cr**	0.016	0.016	0.946	0.065	0.159	0.260
**Mn**	0.020	0.095	0.792	0.205	0.282	0.194
**Fe**	0.005	0.221	25.30	0.728	2.604	5.736
Co *	0.012	0.023	0.023	0.023	0.023	-
**Ni**	0.019	0.053	2.816	0.131	0.320	0.620
**Cu**	0.010	0.065	0.295	0.102	0.121	0.052
**Zn**	0.017	0.253	1.358	0.533	0.607	0.306
**As**	0.015	nd.	nd.	-	-	-
**Se**	0.028	nd.	nd.	-	-	-
**Mo**	0.018	0.025	0.074	0.054	0.051	0.025
**Ag**	0.011	nd.	nd.	-	-	-
**Cd**	0.010	nd.	nd.	-	-	-
**Ba**	0.010	0.012	0.179	0.019	0.031	0.036
Pb *	0.010	0.019	0.019	0.019	0.019	-
**Hg**	0.001	0.001	0.001	0.001	0.001	-
Comb honey (mg/kg)						
**Element**	**LOQ**	**Min**	**Max**	**Median**	**Mean**	**Std Dev**
**Al**	0.025	1.337	21.39	2.978	4.672	4.600
**V**	0.027	0.027	0.053	0.032	0.036	0.012
**Cr**	0.016	0.016	0.415	0.044	0.077	0.080
**Mn**	0.020	0.194	14.88	0.597	2.045	3.416
**Fe**	0.005	0.198	17.16	4.562	5.924	4.613
**Co**	0.012	0.015	0.025	0.021	0.021	0.004
**Ni**	0.019	0.062	0.671	0.204	0.269	0.181
**Cu**	0.010	0.021	2.982	0.207	0.415	0.594
**Zn**	0.017	0.266	32.97	1.859	4.186	6.712
**As**	0.015	nd.	nd.	-	-	-
**Se**	0.028	nd.	nd.	-	-	-
**Mo**	0.018	0.018	0.105	0.027	0.042	0.029
**Ag**	0.011	nd.	nd.	-	-	-
**Cd**	0.010	0.016	0.023	0.022	0.021	0.003
**Ba**	0.010	0.023	0.714	0.074	0.157	0.170
**Pb**	0.010	0.010	0.088	0.021	0.027	0.020
**Hg**	0.001	0.001	0.003	0.001	0.001	0.001

nd.—not detected; * single data point.

## Data Availability

The datasets generated for this study are available on request to the corresponding author.

## Supplementary Materials

The following supporting information can be downloaded at: https://www.mdpi.com/article/10.3390/foods15091502/s1, Table S1. The coordinates of the sampling apiaries and the corresponding code names for the collected honey and comb honey samples. Table S2. List of pesticides analyzed in honey and comb honey samples. Table S3. Validation results for pesticide determinations in honey by LC-MS/MS and GC-MS/MS. Table S4. Validation results for metal determination in honey by ICP-MS. Table S5. Optimized instrumental conditions for ICP-MS Agilent 7900. Table S6. Pesticides identified in honey samples and their concentrations obtained. Table S7. Pesticides identified in comb honey samples and their concentrations obtained.
