# Supplementary material for: Pesticides and Trace Element Residues in Honey from Northern Croatia
Source: Foods. 2026 Apr 25;15(9):1502. doi: 10.3390/foods15091502 (PMC13163511; doi:10.3390/foods15091502)
Supplement: Supplementary file 1 [file foods-15-01502-s001.zip › foods-4267833-supplementary.pdf]

Table S1. The coordinates of the sampling apiaries and the corresponding code names for the collected honey and comb honey samples.

<table>
<tr><th>County</th><th>Location</th><th>Latitude</th><th>Longitude</th><th>Sample code
HONEY
(n =22)</th><th>Sample code
COMB HONEY
(n=38)</th></tr>
<tr><td rowspan="28">Međimurje</td><td rowspan="2">1</td><td rowspan="2">46.43098143</td><td rowspan="2">16.57209587</td><td rowspan="2">1 HONEY 1</td><td>1 COMB HONEY 1</td></tr>
<tr><td>1 COMB HONEY 2</td></tr>
<tr><td rowspan="3">2</td><td rowspan="3">46.45103</td><td rowspan="3">16.63566</td><td rowspan="3">2 HONEY 1</td><td>2 COMB HONEY 1</td></tr>
<tr><td>2 COMB HONEY 2</td></tr>
<tr><td>2 COMB HONEY 3</td></tr>
<tr><td rowspan="2">3</td><td rowspan="2">46.452005</td><td rowspan="2">16.362116</td><td rowspan="2">3 HONEY 1</td><td>3 COMB HONEY 1</td></tr>
<tr><td>3 COMB HONEY 2</td></tr>
<tr><td>4</td><td>46.42634614</td><td>16.55966195</td><td>4 HONEY 1</td><td>4 COMB HONEY 1</td></tr>
<tr><td>5</td><td>46.444619</td><td>16.644286</td><td>5 HONEY 1</td><td>5 COMB HONEY 1</td></tr>
<tr><td rowspan="2">6</td><td rowspan="2">46.444619</td><td rowspan="2">16.644286</td><td rowspan="2">6 HONEY 1</td><td>6 COMB HONEY 1</td></tr>
<tr><td>6 COMB HONEY 2</td></tr>
<tr><td>7</td><td>46.382084</td><td>16.568415</td><td>7 HONEY 2</td><td rowspan="3">7 COMB HONEY 1</td></tr>
<tr><td>7</td><td>46.382084</td><td>16.568415</td><td>7 HONEY 3</td></tr>
<tr><td>7</td><td>46.382084</td><td>16.568415</td><td>7 HONEY 4</td></tr>
<tr><td>8</td><td>46.499402</td><td>16.373567</td><td>8 HONEY 1</td><td>8 COMB HONEY 1</td></tr>
<tr><td rowspan="3">9</td><td rowspan="3">46.47471</td><td rowspan="3">16.512</td><td rowspan="3">9 HONEY 1</td><td>9 COMB HONEY 1</td></tr>
<tr><td>9 COMB HONEY 2</td></tr>
<tr><td>9 COMB HONEY 3</td></tr>
<tr><td rowspan="3">10</td><td rowspan="3">46.38071</td><td rowspan="3">16.51664</td><td rowspan="3">10 HONEY 1</td><td>10 COMB HONEY 1</td></tr>
<tr><td>10 COMB HONEY 2</td></tr>
<tr><td>10 COMB HONEY 3</td></tr>
<tr><td rowspan="2">12</td><td rowspan="2">46.7741249</td><td rowspan="2">16.3640429</td><td rowspan="2">12 HONEY 1</td><td>12 COMB HONEY 1</td></tr>
<tr><td>12 COMB HONEY 2</td></tr>
<tr><td>13</td><td>46.44924</td><td>16.50500943</td><td>13 HONEY 1</td><td>13 COMB HONEY 1</td></tr>
<tr><td>14</td><td>46.44723346</td><td>16.52886182</td><td>14 HONEY 1</td><td>14 COMB HONEY 1</td></tr>
<tr><td rowspan="16">Varaždin</td><td rowspan="3">15</td><td rowspan="3">46.27496699</td><td rowspan="3">16.31527765</td><td rowspan="3">15 HONEY 1</td><td>15 COMB HONEY 1</td></tr>
<tr><td>15 COMB HONEY 2</td></tr>
<tr><td>15 COMB HONEY 3</td></tr>
<tr><td rowspan="3">16</td><td rowspan="3">46.32781208</td><td rowspan="3">16.35647706</td><td rowspan="3">16 HONEY 1</td><td>16 COMB HONEY 4</td></tr>
<tr><td>16 COMB HONEY 18</td></tr>
<tr><td>16 COMB HONEY 36</td></tr>
<tr><td rowspan="3">17</td><td rowspan="3">46.25905738</td><td rowspan="3">16.16136462</td><td rowspan="3">17 HONEY 1</td><td>17 COMB HONEY 2</td></tr>
<tr><td>17 COMB HONEY 22</td></tr>
<tr><td>17 COMB HONEY 33</td></tr>
<tr><td>18</td><td>46.27968197</td><td>16.37522403</td><td>18 HONEY 1</td><td>18 COMB HONEY 1</td></tr>
<tr><td>19</td><td>46.24982901</td><td>16.29542274</td><td>19 HONEY 1</td><td>-</td></tr>
<tr><td rowspan="2">20</td><td rowspan="2">46.24982901</td><td rowspan="2">16.29542274</td><td rowspan="2">20 HONEY 1</td><td>20 COMB HONEY 1</td></tr>
<tr><td>20 COMB HONEY 2</td></tr>
<tr><td rowspan="3">21</td><td rowspan="3">46.2564936</td><td rowspan="3">16.1384275</td><td rowspan="3">21 HONEY 1</td><td>21 COMB HONEY 1</td></tr>
<tr><td>21 COMB HONEY 2</td></tr>
<tr><td>21 COMB HONEY 3</td></tr>
</table>

Table S2. List of pesticides analyzed in honey and comb honey samples

Name	CAS	Mode of action	Chemical group	Instrument	MRL (mg/kg)	Approval (EU pesticides database) Reg.(EC) No 1107/2009
**Acetamiprid**	135410-20-7	insecticide	neonicotinoid	LC-MS/MS	0.05*	approved
**Aldrin**	309-00-2	insecticide	organochlorine	GC-MS/MS	0.01	not approved
**Allethrin**	584-79-2	insecticide	pyrethroid	GC-MS/MS	-	not approved
**Amitraz**	33089-61-1	acaricide, insecticide	amidine	LC-MS/MS	0.2	not approved
**Azinphos-ethyl**	2642-71-9	acaricide, insecticide	organophosphorous	GC-MS/MS	-	not approved
**Azinphos-methyl**	86-50-0	insecticide	organophosphorous	GC-MS/MS	0.05	not approved
**Azoxystrobin**	131860-33-8	fungicide	strobilurin	LC-MS/MS	0.05*	approved
**Benfuracarb**	82560-54-1	nematicide, insecticide	carbamate	LC-MS/MS	0.05*	not approved
**Benzovindiflupyr**	1072957-71-1	fungicide	pyrazolecarboxamide	LC-MS/MS	0.05*	approved
**Bifenthrin (sum of isomers)**	82657-04-3	-	quinoline	GC-MS/MS	0.05*	not approved
**Bixafen**	581809-46-3	fungicide	pyrazole	LC-MS/MS	0.05*	approved
**Boscalid**	188425-85-6	fungicide	pyridinecarboxamide	LC-MS/MS	0.15	approved
**Bromophos-ethyl**	4824-78-6	insecticide	organophosphorous	GC-MS/MS	0.05*	not approved
**Bromopropylate**	18181-80-1	acaricide	benzilate	GC-MS/MS	0.05*	not approved
**Buprofezin**	69327-76-0	-	quinoline	GC-MS/MS	0.05*	approved
**Cadusafos**	95465-99-9	nematicide, insecticide	organophosphorous	GC-MS/MS	0.01*	not approved
**Carbaryl**	63-25-2	growth regulator, insecticide, acaricide	carbamate	GC-MS/MS	0.05*	not approved
**Carbendazim**	10605-21-7	fungicide	benzimidazole	LC-MS/MS	1	not approved
**Carbetamide**	16118-49-3	herbicide	carbamate	LC-MS/MS	0.05*	not approved
**Carbofuran**	1563-66-2	insecticide, acaricide, nematicide	carbamate, N-methyl	LC-MS/MS	0.05*	not approved
**Carbofuran 3-hydroxy**	16655-82-6	-	carbamate, N-methyl	LC-MS/MS	0.05	not approved
**Carbophenothion**	786-19-6	insecticide, acaricide	organophosphorous	GC-MS/MS	0.05*	not approved
**Carbosulfan**	55285-14-8	insecticide	carbamate	LC-MS/MS	0.05*	not approved
**Chlorantraniliprole**	500008-45-7	insecticide	pyrazole	LC-MS/MS	0.05*	approved
**Chlordane, cis-**	5103-71-9	insecticide	organochlorine	GC-MS/MS	0.01	not approved
**Chlordane, trans-**	5103-74-2	insecticide	organochlorine	GC-MS/MS	0.01	not approved
**Chlorfenvinphos**	470-90-6	acaricide, insecticide	organophosphorous	GC-MS/MS	0.01*	not approved
**Chlorobenzilate**	510-15-6	acaricide	organochlorine	GC-MS/MS	-	not approved
**Chlorpropham**	101-21-3	herbicide, growth regulator	carbamate	LC-MS/MS	0.05*	not approved
**Chlorpyrifos**	2921-88-2	insecticide	organophosphorous	GC-MS/MS	0.01*	not approved
**Chlorpyrifos-methyl**	5598-13-0	insecticide, acaricide	organophosphorous	GC-MS/MS	0.01*	not approved
**Clothianidin**	210880-92-5	insecticide	neonicotinoid	LC-MS/MS	0.05*	not approved
**Coumaphos**	56-72-4	insecticide	phosphorothiolate	LC-MS/MS	0.1	not approved
**Cyfluthrin (sum of isomers)**	68359-37-5	insecticide	pyrethroid	GC-MS/MS	0.05*	not approved
**Cymiazol**	61676-87-7	-	quinoline	LC-MS/MS	-	
**Cypermethrin (sum of isomers)**	52315-07-8	insecticide	pyrethroid	GC-MS/MS	0.05*	approved
**Cyproconazol**	94361-06-5	fungicide	triazole	LC-MS/MS	0.05*	not approved
**Cyprodinil**	121552-61-2	fungicide	anilinopyrimidine	LC-MS/MS	0.05*	approved
**DDD-p,p'**	72-54-8	insecticide	organochlorine	GC-MS/MS	0.05	not approved
**DDE-p,p'**	72-55-9	insecticide	organochlorine	GC-MS/MS	0.05	not approved

**DDT-o,p'**	789-02-6	insecticide	organochlorine	GC-MS/MS	0.05	not approved
**DDT-p,p'**	50-29-3	insecticide	organochlorine	GC-MS/MS	0.05	not approved
**DEET (Diethyl-m-toluamide, N,N-)**	134-62-3	insecticide	-	GC-MS/MS	-	
**Deltamethrin**	52918-63-5	insecticide	pyrethroid	LC-MS/MS	0.05*	approved
**Demeton-S-methyl**	919-86-8	insecticide, acaricide	organophosphorous	GC-MS/MS	-	not approved
**Demeton-S-methylsulfone**	17040-19-6	acaricide, insecticide	organophosphorous	GC-MS/MS	0.01*	not approved
**Demeton-S-methyl-sulfoxide (Oxydemethon-methyl)**	301-12-2	insecticide	organophosphorous	LC-MS/MS	0.01*	not approved
**Diazinon**	333-41-5	acaricide, insecticide, nematicide	organophosphorous	GC-MS/MS	0.01*	not approved
**Dichlorvos**	62-73-7	insecticide, acaricide	organophosphorous	LC-MS/MS	-	not approved
**Dieldrin**	60-57-1	insecticide	organochlorine	GC-MS/MS	0.01	not approved
**Difenoconazole**	119446-68-3	fungicide	triazole	LC-MS/MS	0.05*	approved
**Dimethoate**	60-51-5	acaricide, insecticide	organophosphorous	LC-MS/MS	0.01*	not approved
**Dimethomorph (sum of isomers)**	110488-70-5	fungicide	morpholine	LC-MS/MS	0.05*	not approved
**Dimoxystrobin**	149961-52-4	fungicide	strobilurin	LC-MS/MS	0.05*	not approved
**Dinotefuran**	165252-70-0	insecticide	neonicotinoid	LC-MS/MS	-	not approved
**Diphenylamine**	122-39-4	fungicide	-	GC-MS/MS	0.05*	not approved
**Disulfoton**	298-04-4	-	organophosphorous	GC-MS/MS	0.01*	not approved
**Disulfoton-sulfone**	2497-06-5	-	organophosphorous	GC-MS/MS	0.01*	not approved
**Disulfoton-sulfoxide**	2497-07-6	-	organophosphorous	GC-MS/MS	0.01*	not approved
**Endosulfan, alpha-**	959-98-8	acaricide, insecticide	organochlorine	GC-MS/MS	0.01*	not approved
**Endosulfan, beta-**	33213-65-9	acaricide, insecticide	organochlorine	GC-MS/MS	0.01*	not approved
**Endosulfansulfate**	1031-07-8	acaricide, insecticide	organochlorine	GC-MS/MS	0.01*	not approved
**Endrin**	72-20-8	insecticide	organochlorine	GC-MS/MS	0.01	not approved
**Epoxiconazole**	133855-98-8	fungicide	triazole	LC-MS/MS	0.05*	not approved
**Ethion**	563-12-2	acaricide, insecticide	organophosphorous	GC-MS/MS	0.01*	not approved
**Ethoprophos**	13194-48-4	insecticide, nematicide	organophosphorous	LC-MS/MS	0.05*	not approved
**Etofenprox**	80844-07-1	insecticide	pyrethroid, non-ester	LC-MS/MS	0.05*	approved
**Famoxadone**	131807-57-3	fungicide	strobilurin	LC-MS/MS	0.05*	not approved
**Fenchlorphos**	299-84-3	insecticide	organophosphorous	GC-MS/MS	-	not approved
**Fenchlorphos-oxon**	3983-45-7	-	organophosphorous	GC-MS/MS	-	not approved
**Fenhexamid**	126833-17-8	fungicide	hydroxyanilide	LC-MS/MS	0.05*	approved
**Fenitrothion**	122-14-5	insecticide	organophosphorous	GC-MS/MS	0.01*	not approved
**Fenoxycarb**	72490-01-8	insecticide	carbamate	LC-MS/MS	0.05*	not approved
**Fenpropathrin**	39515-41-8	acaricide, insecticide	pyrethroid	GC-MS/MS	-	not approved
**Fenpropidin**	67306-00-7	fungicide	morpholine	LC-MS/MS	0.05*	approved
**Fenpropimorph**	67564-91-4	fungicide	morpholine	LC-MS/MS	0.05*	not approved
**Fenpyrazamine**	473798-59-3	fungicide	pyrazole	LC-MS/MS	0.05*	not approved
**Fensulfothion**	115-90-2	insecticide, nematicide	organophosphorous	GC-MS/MS	-	not approved
**Fensulfothion-oxon**	6552-21-2	-	organophosphorous	GC-MS/MS	-	not approved
**Fensulfothion-oxon-sulfon**	6132-17-8	-	metabolite	GC-MS/MS	-	not approved
**Fensulfothion-sulfone**	14255-72-2	-	organophosphorous	GC-MS/MS	-	not approved
**Fenthion**	55-38-9	insecticide	organophosphorous	LC-MS/MS	0.01*	not approved
**Fenthion-oxon**	6552-12-1	-	organophosphorous	LC-MS/MS	0.01*	not approved

**Fenthion-oxon-sulfone**	14086-35-2	-	organophosphorous	LC-MS/MS	0.01*	not approved
**Fenthion-oxon-sulfoxide**	6552-13-2	-	organophosphorous	LC-MS/MS	0.01*	not approved
**Fenthion-sulfone**	3761-42-0	-	organophosphorous	LC-MS/MS	0.01*	not approved
**Fenthion-sulfoxide**	3761-41-9	-	organophosphorous	LC-MS/MS	0.01*	not approved
**Fenvalerate**	51630-58-1	insecticide, acaricide	pyrethroid	GC-MS/MS	0.05*	not approved
**Fipronil**	120068-37-3	insecticide	phenylpyrazole	LC-MS/MS	0.005*	not approved
**Fipronil-desulfinyl**	205650-65-3	-	metabolite	LC-MS/MS	-	not approved
**Fipronil-sulfide**	120067-83-6	-	metabolite	LC-MS/MS	-	not approved
**Fipronil-sulfone**	120068-36-2	-	metabolite	LC-MS/MS	0.005*	not approved
**Fluopyram**	658066-35-4	fungicide	benzamide	LC-MS/MS	0.05*	approved
**Fluopyram-benzamide**	360-64-5	-	metabolite	LC-MS/MS	0.05*	approved
**Fluquinconazole**	136426-54-5	fungicide	triazole	LC-MS/MS	0.05*	not approved
**Flusilazole**	85509-19-9	fungicide	triazole	LC-MS/MS	0.05*	not approved
**Flutriafol**	76674-21-0	fungicide	triazole	LC-MS/MS	0.05*	not approved
**Fluvalinate,tau**	102851-06-9	acaricide, insecticide	pyrethroid	LC-MS/MS	0.05*	approved
**Fluxapyroxad**	907204-31-3	fungicide	pyrazole	LC-MS/MS	0.05*	approved
**Furathiocarb**	65907-30-4	insecticide	carbamate	GC-MS/MS	0.05*	not approved
**Glufosinate**	51276-47-2	fungicide	phosphinic acid	LC-MS/MS	0.05*	not approved
**Glyphosate**	1071-83-6	herbicide	glycine derivative	LC-MS/MS	0.05*	approved
**HCH, alpha-**	319-84-6	insecticide	organochlorine	GC-MS/MS	0.01*	not approved
**HCH, beta-**	319-85-7	insecticide	organochlorine	GC-MS/MS	0.01*	not approved
**HCH, gamma-/Lindan**	58-89-9	insecticide	organochlorine	GC-MS/MS	0.01*	not approved
**Heptachlor**	76-44-8	insecticide	organochlorine	GC-MS/MS	0.01	not approved
**Heptachlorepoxid, egzo-**	1024-57-3	-	organochlorine	GC-MS/MS	0.01	not approved
**Heptachlorepoxid, endo-**	28044-83-9	-	organochlorine	GC-MS/MS	0.01	not approved
**Heptenophos**	23560-59-0	insecticide	organophosphorous	LC-MS/MS	-	not approved
**Hexachlorobenzene**	118-74-1	fungicide	organochlorine	GC-MS/MS	0.01*	not approved
**Hexythiazox**	78587-05-0	acaricide, insecticide	-	LC-MS/MS	0.05*	approved
**Imazalil**	35554-44-0	fungicide	imidazole	LC-MS/MS	0.05*	approved
**Imidacloprid**	138261-41-3	insecticide	neonicotinoid	LC-MS/MS	0.05*	not approved
**Indoxacarb**	173584-44-6	insecticide	oxadiazine	LC-MS/MS	0.05*	not approved
**Iprodione**	36734-19-7	fungicide	dicarboximide	LC-MS/MS	0.05*	not approved
**Kresoxim-methyl**	143390-89-0	fungicide	strobilurin	LC-MS/MS	0.05*	approved
**lambda-Cyhalothrin**	91465-08-6	insecticide	pyrethroid	GC-MS/MS	0.05*	approved
**Malaoxon**	1634-78-2	-	organophosphorous	LC-MS/MS	0.05*	approved
**Malathion**	121-75-5	insecticide, acaricide	organophosphorous	GC-MS/MS	0.05*	approved
**Metaflumizone**	139968-49-3	insecticide	-	LC-MS/MS	0.05*	not approved
**Methacrifos**	62610-77-9	acaricide, insecticide	organophosphorous	GC-MS/MS	0.05*	not approved
**Methidathion**	950-37-8	acaricide, insecticide	organophosphorous	GC-MS/MS	0.02*	not approved
**Methiocarb**	2032-65-7	acaricide, molluscicide, repellent, insecticide	carbamate, N-methyl	LC-MS/MS	0.05*	not approved
**Methiocarb-sulfone**	2179-25-1	-	carbamate, N-methyl	LC-MS/MS	0.05*	not approved
**Methiocarb-sulfoxide**	2635-10-1	-	carbamate, N-methyl	LC-MS/MS	0.05*	not approved
**Methoxychlor**	72-43-5	insecticide	organochlorine	GC-MS/MS	-	not approved
**Mevinphos**	7786-34-7	insecticide, acaricide	organophosphorous	GC-MS/MS	0.05*	not approved
**Myclobutanil**	88671-89-0	fungicide	triazole	LC-MS/MS	0.05*	not approved
**N-(2,4 dimethylphenyl)formamide (DMF)**	60397-77-5	-	metabolite	LC-MS/MS	0.2	not approved

**N-(2,4 dimethylphenyl)-N`-methylformamidine (DMPF)**	33089-74-6	-	metabolite	LC-MS/MS	0.2	not approved
**N-Acetyl AMPA**	57637-97-5	-	-	LC-MS/MS	0.05*	approved
**Nitrofen**	1836-75-5	herbicide	organochlorine	GC-MS/MS	-	not approved
**Omethoate**	1113-02-6	insecticide, acaricide	organophosphorous	LC-MS/MS	0.01*	not approved
**Oxychlordane**	27304-13-8	-	organochlorine	GC-MS/MS	0.01	not approved
**Oxyfluorfen**	42874-03-3	herbicide	diphenyl ether	GC-MS/MS	0.05*	approved
**Paraoxon-methyl**	950-35-6	-	organophosphorous	LC-MS/MS	0.01*	not approved
**Parathion**	56-38-2	insecticide, acaricide	organophosphorous	GC-MS/MS	-	not approved
**Parathion-methyl**	298-00-0	insecticide	organophosphorous	GC-MS/MS	0.01*	not approved
**Pendimethalin**	40487-42-1	herbicide	dinitroaniline	LC-MS/MS	0.05*	approved
**Penflufen**	494793-67-8	fungicide	pyrazole	LC-MS/MS	0.05*	not approved
**Pentachloroaniline**	527-20-8	-	organochlorine	GC-MS/MS	0.01*	not approved
**Penthiopyrad**	183675-82-3	fungicide	pyrazolecarboxamide	LC-MS/MS	0.05*	approved
**Permethrin (sum of isomers)**	52645-53-1	insecticide	pyrethroid	GC-MS/MS	-	not approved
**Phenylphenol, 2-**	90-43-7	fungicide	-	GC-MS/MS	0.05*	approved
**Phosalone**	2310-17-0	insecticide, acaricide	organophosphorous	LC-MS/MS	0.05*	not approved
**Phosmet**	732-11-6	acaricide, insecticide	organophosphorous	LC-MS/MS	0.05*	not approved
**Phoxim**	14816-18-3	insecticide	organophosphorous	LC-MS/MS	0.02*	not approved
**Piperonyl butoxide**	51-03-6	-	-	GC-MS/MS	-	Not in the scope of Directive 91/414/EEC (not a PPP)
**Pirimicarb**	23103-98-2	insecticide	carbamate	GC-MS/MS	0.05*	approved
**Pirimicarb-desmethyl**	30614-22-3	-	carbamate	LC-MS/MS	0.05*	approved
**Pirimiphos-ethyl**	23505-41-1	insecticide	organophosphorous	GC-MS/MS	-	not approved
**Pirimiphos-methyl**	29232-93-7	insecticide, acaricide	organophosphorous	GC-MS/MS	0.05*	approved
**Prochloraz**	67747-09-5	fungicide	imidazole	LC-MS/MS	0.15	not approved
**Procymidone**	32809-16-8	fungicide	dicarboximide	GC-MS/MS	0.05*	not approved
**Profenofos**	41198-08-7	insecticide, acaricide	organophosphorous	GC-MS/MS	0.05*	not approved
**Propargite**	2312-35-8	acaricide	-	LC-MS/MS	0.05*	not approved
**Propetamphos**	31218-83-4	acaricide, insecticide	organophosphorous	GC-MS/MS	-	not approved
**Propyzamide**	23950-58-5	herbicide	benzamide	LC-MS/MS	0.05*	approved
**Prosulfocarb**	52888-80-9	herbicide	thiocarbamate	GC-MS/MS	0.05*	approved
**Prothioconazole-desthio**	120983-64-4	-	triazole	GC-MS/MS	0.05*	approved
**Pyraclostrobin**	175013-18-0	fungicide	strobilurin	LC-MS/MS	0.05*	approved
**Pyrazophos**	13457-18-6	fungicide	phosphorothiolate	GC-MS/MS	0.05*	not approved
**Pyrimethanil**	53112-28-0	fungicide	anilinopyrimidine	LC-MS/MS	0.3	approved
**Quinalphos**	13593-03-8	acaricide, insecticide	organophosphorous	GC-MS/MS	0.05*	not approved
**Quintozen**	82-68-8	fungicide	organochlorine	GC-MS/MS	0.01*	not approved
**Resmethrin**	10453-86-8	insecticide	pyrethroid	LC-MS/MS	0.05*	not approved
**Spinosyn-A**	131929-60-7	insecticide	spinosyn	LC-MS/MS	0.05*	approved
**Spinosyn-D**	131929-63-0	insecticide	spinosyn	LC-MS/MS	0.05*	approved
**Spiroxamine**	118134-30-8	fungicide	morpholine	LC-MS/MS	0.05*	approved
**Sulfoxaflor**	946578-00-3	insecticide	sulfoximine	LC-MS/MS	0.05*	approved
**Tebuconazole**	107534-96-3	fungicide	triazole	LC-MS/MS	0.05*	approved
**Tebufenozide**	112410-23-8	insecticide	diacylhydrazine	LC-MS/MS	0.05*	approved
**Tebufenpyrad**	119168-77-3	acaricide	pyrazole	LC-MS/MS	0.05*	approved
**Tecnazene**	117-18-0	fungicide, growth regulator	aromatic hydrocarbon	GC-MS/MS	0.05*	not approved
**Terbufos**	13071-79-9	insecticide, nematicide	organophosphorous	GC-MS/MS	-	not approved

**Terbufos-sulfone**	56070-16-7	-	organophosphorous	GC-MS/MS	-	not approved
**Terbufos-sulfoxide**	10548-10-4	-	organophosphorous	GC-MS/MS	-	not approved
**Terbuthylazine**	5915-41-3	herbicide	triazine	LC-MS/MS	0.05*	approved
**Tetrachlorvinphos**	22248-79-9	acaricide, insecticide	organophosphorous	GC-MS/MS	-	not approved
**Tetraconazole**	112281-77-3	fungicide	triazole	LC-MS/MS	0.05*	approved
**Tetramethrin**	7696-12-0	insecticide	pyrethroid	GC-MS/MS	-	not approved
**Thiacloprid**	111988-49-9	insecticide	neonicotinoid	LC-MS/MS	0.2	not approved
**Thiamethoxam**	153719-23-4	insecticide	neonicotinoid	LC-MS/MS	0.05*	not approved
**Thiophanate-methyl**	23564-05-8	fungicide	benzimidazole	LC-MS/MS	1	not approved
**Tolclofos-methyl**	57018-04-9	fungicide	aromatic hydrocarbon	GC-MS/MS	0.05*	approved
**Triazophos**	24017-47-8	nematicide, insecticide, acaricide	organophosphorous	GC-MS/MS	0.05*	not approved
**Trichlorfon**	52-68-6	insecticide	organophosphorous	LC-MS/MS	0.01*	not approved
**Trifloxystrobin**	141517-21-7	fungicide	strobilurin	LC-MS/MS	0.07	approved
**Trifluralin**	1582-09-8	herbicide	dinitroaniline	GC-MS/MS	0.05*	not approved
**Vinclozolin**	50471-44-8	fungicide	dicarboximide	GC-MS/MS	0.05*	not approved

Table S3. Validation results for pesticide determinations in honey by LC-MS/MS and GC-MS/MS

Name	LOQ (mg/kg)	Linearity range (mg/kg)	R^2	Recovery, % (RSD_r, %)				
				0.001 mg/kg	***0.002 mg/kg***	***0.005 mg/kg***	***0.01 mg/kg***	***0.02 mg/kg***
**Acetamiprid**	0.001	0.001-0.25	0.985	83 (11)	97 (9)	91 (9)	110 (6)	101 (11)
**Aldrin**	0.001	0.001-0.25	0.993	92 (8)	85 (9)	86 (10)	96 (7)	99 (12)
**Allethrin**	0.01	0.01-0.25	0.980	-	-	-	71 (12)	103 (8)
**Amitraz**	0.001	0.001-0.25	0.986	94 (7)	96 (12)	102 (14)	93 (8)	91 (7)
**Azinphos-ethyl**	0.001	0.001-0.25	0.993	90 (18)	85 (10)	92 (5)	90 (11)	87 (13)
**Azinphos-methyl**	0.001	0.001-0.25	0.991	88 (13)	76 (8)	106 (7)	101 (12)	98 (5)
**Azoxystrobin**	0.001	0.001-0.25	0.997	78 (10)	85 (5)	80 (12)	87 (4)	89 (3)
**Benfuracarb**	0.001	0.001-0.25	0.986	89 (20)	96 (4)	93 (14)	85 (7)	88 (6)
**Benzovindiflupyr**	0.001	0.001-0.25	0.990	91 (14)	109 (12)	71 (7)	89 (4)	96 (10)
**Bifenthrin (sum of isomers)**	0.005	0.005-0.25	0.982	-	-	79 (14)	106 (9)	97 (7)
**Bixafen**	0.001	0.001-0.25	0.985	90 (9)	111 (4)	88 (6)	92 (6)	90 (9)
**Boscalid**	0.001	0.001-0.25	0.979	106 (11)	100 (7)	82 (6)	99 (13)	98 (16)
**Bromophos-ethyl**	0.001	0.001-0.25	0.986	88 (13)	91 (13)	107 (14)	92 (7)	90 (4)
**Bromopropylate**	0.001	0.001-0.25	0.979	97 (6)	87 (8)	91 (9)	97 (10)	99 (12)
**Buprofezin**	0.001	0.001-0.25	0.963	110 (12)	85 (10)	102 (13)	111 (5)	94 (4)
**Cadusafos**	0.001	0.001-0.25	0.989	88 (10)	89 (7)	87 (18)	97 (7)	96 (3)
**Carbaryl**	0.005	0.005-0.25	0.982	-	-	91 (8)	95 (6)	116 (11)
**Carbendazim**	0.001	0.001-0.25	0.999	103 (17)	107 (10)	89 (4)	99 (3)	96 (9)
**Carbetamide**	0.001	0.001-0.25	0.986	89 (8)	108 (9)	85 (5)	83 (11)	86 (5)
**Carbofuran**	0.001	0.001-0.25	0.994	92 (11)	91 (7)	72 (6)	88 (3)	92 (8)
**Carbofuran 3-hydroxy**	0.005	0.005-0.25	0.983	-	-	105 (11)	103 (8)	96 (4)
**Carbophenothion**	0.001	0.001-0.25	0.987	86 (12)	99 (8)	99 (10)	107 (7)	92 (3)
**Carbosulfan**	0.001	0.001-0.25	0.989	77 (16)	101 (6)	94 (13)	75 (16)	106 (11)
**Chlorantraniliprole**	0.002	0.002-0.25	0.999	-	102 (7)	106 (3)	89 (4)	89 (6)
**Chlordane, cis-**	0.001	0.001-0.25	0.979	81 (13)	87 (10)	89 (7)	109 (10)	92 (11)
**Chlordane, trans-**	0.001	0.001-0.25	0.981	77 (8)	91 (6)	93 (9)	107 (11)	98 (6)
**Chlorfenvinphos**	0.001	0.001-0.25	0.974	90 (12)	112 (7)	91 (6)	72 (8)	81 (9)
**Chlorobenzilate**	0.001	0.001-0.25	0.986	72 (14)	87 (10)	86 (12)	79 (13)	97 (9)
**Chlorpropham**	0.005	0.005-0.25	0.999	-	-	99 (16)	97 (11)	97 (4)
**Chlorpyrifos**	0.001	0.001-0.25	0.980	86 (10)	70 (8)	71 (10)	92 (14)	96 (10)
**Chlorpyrifos-methyl**	0.001	0.001-0.25	0.976	104 (11)	99 (7)	104 (10)	83 (18)	106 (3)

**Clothianidin**	0.002	0.002-0.25	0.987	-	101 (8)	101 (3)	99 (6)	98 (4)
**Coumaphos**	0.001	0.001-0.25	1.000	96 (15)	100 (8)	99 (7)	105 (5)	102 (6)
**Cyfluthrin (sum of isomers)**	0.001	0.001-0.25	0.967	72 (19)	78 (6)	76 (7)	90 (11)	93 (12)
**Cymiazol**	0.001	0.001-0.25	0.994	116 (9)	79 (4)	81 (2)	89 (6)	74 (5)
**Cypermethrin (sum of isomers)**	0.01	0.01-0.25	0.970	-	-	-	80 (10)	91 (7)
**Cyproconazol**	0.001	0.001-0.25	0.992	95 (17)	96 (4)	78 (6)	109 (2)	82 (18)
**Cyprodinil**	0.001	0.001-0.25	0.985	97 (12)	93 (15)	105 (12)	102 (11)	100 (8)
**DDD-p,p'**	0.001	0.001-0.25	0.982	89 (6)	99 (4)	87 (14)	96 (6)	85 (10)
**DDE-p,p'**	0.001	0.001-0.25	0.976	91 (9)	106 (2)	111 (9)	91 (2)	103 (9)
**DDT-o,p'**	0.001	0.001-0.25	0.977	78 (13)	89 (7)	105 (7)	89 (3)	96 (4)
**DDT-p,p'**	0.001	0.001-0.25	0.990	82 (10)	105 (16)	109 (5)	78 (10)	95 (3)
**DEET (Diethyl-m-toluamide, N,N-)**	0.005	0.005-0.25	0.986	-	-	77 (5)	98 (3)	96 (7)
**Deltamethrin**	0.005	0.005-0.25	0.987	-	-	81 (13)	90 (11)	91 (10)
**Demeton-S-methyl**	0.005	0.005-0.25	0.982	-	-	87 (12)	96 (16)	102 (11)
**Demeton-S-methylsulfone**	0.005	0.005-0.25	0.981	-	-	102 (18)	90 (12)	107 (8)
**Demeton-S-methyl-sulfoxide (Oxydemethon-methyl)**	0.001	0.001-0.25	0.992	70 (11)	77 (10)	96 (9)	99 (13)	107 (9)
**Diazinon**	0.001	0.001-0.25	0.999	80 (13)	97 (9	99 (4)	102 (5)	103 (6)
**Dichlorvos**	0.002	0.002-0.25	0.985	-	102 (12)	97 (12)	76 (14)	108 (10)
**Dieldrin**	0.001	0.001-0.25	0.996	85 (7)	92 (5)	106 (11)	98 (8)	94 (3)
**Difenoconazole**	0.001	0.001-0.25	0.996	86 (5)	96 (7)	103 (10)	95 (6)	98 (8)
**Dimethoate**	0.001	0.001-0.25	0.984	99 (14)	106 (3)	88 (7)	105 (4)	84 (11)
**Dimethomorph (sum of isomers)**	0.001	0.001-0.25	0.981	81 (10)	108 (8)	89 (5)	88 (18)	92 (4)
**Dimoxystrobin**	0.001	0.001-0.25	0.982	92 (5)	101 (6)	96 (12)	89 (15)	91 (7)
**Dinotefuran**	0.005	0.005-0.25	0.992	-	-	87 (10)	86 (10)	78 (10)
**Diphenylamine**	0.001	0.001-0.25	0.984	76 (15)	79 (7)	83 (9)	92 (11)	88 (7)
**Disulfoton**	0.005	0.005-0.25	0.987	-	-	72 (11)	87 (13)	97 (8)
**Disulfoton-sulfone**	0.005	0.005-0.25	0.990	-	-	85 (16)	91 (7)	107 (9)
**Disulfoton-sulfoxide**	0.005	0.005-0.25	0.986	-	-	82 (10)	90 (6)	97 (5)
**Endosulfan, alpha-**	0.001	0.001-0.25	0.991	79 (11)	102 (5)	86 (6)	102 (7)	104 (5)
**Endosulfan, beta-**	0.001	0.001-0.25	0.994	81 (14)	93 (7)	104 (11)	88 (4)	94 (6)
**Endosulfansulfate**	0.001	0.001-0.25	0.987	82 (12)	97 (6)	99 (5)	89 (14)	100 (6)
**Endrin**	0.001	0.001-0.25	0.974	106 (19)	102 (7)	99 (13)	96 (7)	98 (3)
**Epoxiconazole**	0.001	0.001-0.25	0.981	76 (5)	85 (5)	91 (9)	103 (8)	105 (4)
**Ethion**	0.001	0.001-0.25	0.994	72 (16)	81 (6)	92 (11)	85 (15)	104 (13)
**Ethoprophos**	0.001	0.001-0.25	0.998	82 (16)	97 (6)	97 (14)	97 (16)	95 (10)
**Etofenprox**	0.001	0.001-0.25	0.999	93 (10)	107 (11)	101 (19)	92 (6)	113 (9)
**Famoxadone**	0.002	0.002-0.25	0.992	-	84 (15)	81 (10)	96 (5)	99 (6)
**Fenchlorphos**	0.005	0.005-0.25	0.973	-	-	98 (13)	97 (10)	87 (12)
**Fenchlorphos-oxon**	0.005	0.005-0.25	0.971	-	-	112 (5)	108 (9)	94 (9)
**Fenhexamid**	0.005	0.005-0.25	0.982	-	-	99 (6)	86 (14)	90 (6)
**Fenitrothion**	0.001	0.001-0.25	0.967	116 (17)	98 (13)	82 (11)	79 (10)	89 (5)
**Fenoxycarb**	0.001	0.001-0.25	0.994	90 (11)	106 (10)	93 (17)	92 (3)	95 (11)
**Fenpropathrin**	0.001	0.001-0.25	0.986	85 (8)	92 (19)	96 (12)	96 (5)	102 (5)
**Fenpropidin**	0.001	0.001-0.25	0.992	88 (11)	103 (14)	105 (5)	92 (4)	88 (9)
**Fenpropimorph**	0.001	0.001-0.25	0.999	90 (6)	86 (12)	93 (6)	90 (13)	104 (6)
**Fenpyrazamine**	0.001	0.001-0.25	0.987	-	-	96 (15)	107 (7)	90 (7)
**Fensulfothion**	0.005	0.005-0.25	0.998	-	-	71 (20)	74 (15)	77 (5)
**Fensulfothion-oxon**	0.005	0.005-0.25	0.974	-	-	109 (10)	78 (7)	86 (4)

**Fensulfothion-oxon-sulfon**	0.005	0.005-0.25	0.970	-	-	94 (147)	90 (4)	88 (6)
**Fensulfothion-sulfone**	0.005	0.005-0.25	0.968	-	-	79 (12)	81 (15)	83 (12)
**Fenthion**	0.002	0.002-0.25	0.997	-	81 (13)	104 (10)	92 (6)	91 (7)
**Fenthion-oxon**	0.002	0.002-0.25	0.992	-	104 (4)	109 (5)	84 (11)	85 (6)
**Fenthion-oxon-sulfone**	0.002	0.002-0.25	0.994	-	111 (7)	95 (12)	96 (9)	90 (10)
**Fenthion-oxon-sulfoxide**	0.002	0.002-0.25	0.996	-	98 (5)	110 (9)	104 (3)	107 (4)
**Fenthion-sulfone**	0.002	0.002-0.25	0.987	-	92 (14)	80 (8)	91 (11)	105 (5)
**Fenthion-sulfoxide**	0.002	0.002-0.25	0.983	-	92 (16)	93 (14)	88 (16)	72 (12)
**Fenvalerate**	0.002	0.002-0.25	0.981	-	72 (10)	104 (5)	92 (6)	103 (9)
**Fipronil**	0.001	0.001-0.1	0.997	98 (8)	96 (6)	100 (6)	97 (11)	96 (10)
**Fipronil-desulfinyl**	0.001	0.001-0.1	0.999	95 (10)	107 (14)	102 (7)	93 (9)	92 (11)
**Fipronil-sulfide**	0.001	0.001-0.1	0.998	99 (5)	91 (9)	103 (10)	92 (5)	97 (13)
**Fipronil-sulfone**	0.001	0.001-0.1	0.995	96 (11)	102 (13)	99 (5)	95 (10)	101 (7)
**Fluopyram**	0.001	0.001-0.25	0.997	80 (13)	87 (10)	86 (19)	92 (10)	105 (6)
**Fluopyram-benzamide**	0.001	0.001-0.25	0.984	79 (12)	107 (9)	98 (18)	105 (7)	102 (4)
**Fluquinconazole**	0.005	0.005-0.25	0.986	-	-	95 (5)	92 (6)	101 (7)
**Flusilazole**	0.001	0.001-0.25	0.981	86 (11)	89 (18)	109 (10)	107 (7)	92 (3)
**Flutriafol**	0.001	0.001-0.25	0.987	79 (9)	108 (5)	96 (12)	75 (16)	106 (11)
**Fluvalinate,tau**	0.002	0.002-0.25	0.965	-	103 (8)	107 (3)	89 (4)	89 (6)
**Fluxapyroxad**	0.001	0.001-0.25	0.987	89 (5)	88 (12)	89 (7)	109 (10)	92 (11)
**Furathiocarb**	0.001	0.001-0.25	0.997	78 (16)	92 (7)	93 (9)	107 (11)	98 (6)
**HCH, alpha-**	0.001	0.001-0.25	0.986	92 (6)	91 (7)	99 (16)	97 (11)	97 (4)
**HCH, beta-**	0.001	0.001-0.25	0.992	84 (11)	85 (6)	92 (16)	91 (6)	99 (5)
**HCH, gamma-/Lindan**	0.001	0.001-0.25	0.991	103 (12)	93 (8)	90 (7)	104 (11)	103 (8)
**Heptachlor**	0.001	0.001-0.25	0.982	95 (5)	91 (11)	89 (13)	94 (10)	102 (7)
**Heptachlorepoxid, egzo-**	0.001	0.001-0.25	0.987	107 (7)	111 (12)	92 (5)	91 (13)	85 (16)
**Heptachlorepoxid, endo-**	0.001	0.001-0.25	0.986	82 (12)	97 (4)	84 (3)	102 (3)	82 (4)
**Heptenophos**	0.001	0.001-0.25	0.985	95 (14)	95 (7)	87 (6)	82 (7)	101 (10)
**Hexachlorobenzene**	0.001	0.001-0.25	0.996	72 (7)	99 (4)	70 (10)	80 (14)	72 (15)
**Hexythiazox**	0.001	0.001-0.25	0.984	78 (14)	107 (9)	90 (7)	86 (5)	107 (6)
**Imazalil**	0.001	0.001-0.25	0.992	89 (6)	93 (6)	98 (9)	85 (6)	108 (15)
**Imidacloprid**	0.001	0.001-0.25	0.999	106 (18)	105 (10)	104 (16)	102 (8)	101 (7)
**Indoxacarb**	0.002	0.002-0.25	0.993	-	105 (14)	107 (6)	99 (4)	98 (9)
**Iprodione**	0.005	0.005-0.25	0.997	-	-	96 (4)	84 (14)	87 (5)
**Kresoxim-methyl**	0.001	0.001-0.25	0.991	105 (11)	94 (8)	101 (10)	105 (12)	108 (18)
**lambda-Cyhalothrin**	0.005	0.005-0.25	0.996	-	-	92 (17)	102 (5)	108 (11)
**Malaoxon**	0.001	0.001-0.25	0.992	71 (12)	77 (9)	76 (7)	81 (5)	87 (6)
**Malathion**	0.001	0.001-0.25	0.987	89 (12)	102 (5)	86 (10)	95 (13)	116 (3)
**Metaflumizone**	0.002	0.002-0.25	0.994	-	-	92 (8)	87 (6)	104 (16)
**Methacrifos**	0.001	0.001-0.25	0.991	91 (7)	82 (6)	92 (7)	103 (10)	93 (9)
**Methidathion**	0.001	0.001-0.25	0.980	90 (4)	90 (10)	91 (14)	75 (15)	99 (8)
**Methiocarb**	0.001	0.001-0.25	0.979	115 (14)	108 (12)	102 (11)	109 (9)	102 (8)
**Methiocarb-sulfone**	0.001	0.001-0.25	0.993	109 (18)	103 (7)	89 (13)	97 (7)	98 (3)
**Methiocarb-sulfoxide**	0.001	0.001-0.25	0.998	77 (5)	95 (5)	92 (9)	105 (8)	105 (4)
**Methoxychlor**	0.001	0.001-0.25	0.980	82 (16)	91 (6)	96 (11)	87 (15)	105 (13)
**Mevinphos**	0.001	0.001-0.25	0.986	92 (16)	87 (6)	92 (14)	77 (16)	85 (10)
**Myclobutanil**	0.001	0.001-0.25	0.988	94 (10)	117 (11)	105 (19)	72 (6)	103 (9)
**N-(2,4 dimethylphenyl)form amide (DMF)**	0.005	0.005-0.25	0.999	-	-	107 (9)	102 (10)	105 (9)
**N-(2,4 dimethylphenyl)-N`-methylformamidine (DMPF)**	0.005	0.005-0.25	0.994	-	-	103 (6)	105 (7)	73 (9)

**Nitrofen**	0.001	0.001-0.25	0.987	85 (12)	102 (8)	95 (14)	85 (16)	108 (11)
**Omethoate**	0.001	0.001-0.25	0.983	87 (3)	92 (7)	107 (5)	81 (4)	82 (6)
**Oxychlordane**	0.002	0.002-0.25	0.966	-	86 (9)	71 (7)	119 (10)	90 (11)
**Oxyfluorfen**	0.001	0.001-0.25	0.984	77 (10)	92 (7)	83 (9)	97 (11)	91 (6)
**Paraoxon-methyl**	0.001	0.001-0.25	0.986	81 (4)	113 (8)	81 (6)	75 (8)	83 (9)
**Parathion**	0.001	0.001-0.25	0.984	74 (10)	88 (12)	106 (12)	89 (13)	91 (9)
**Parathion-methyl**	0.001	0.001-0.25	0.975	79 (4)	111 (9)	96 (10)	106 (7)	90 (4)
**Pendimethalin**	0.002	0.002-0.25	0.982	-	92 (6)	106 (11)	74 (15)	100 (3)
**Penflufen**	0.001	0.001-0.25	0.996	88 (11)	95 (6)	100 (16)	92 (6)	97 (5)
**Pentachloroaniline**	0.001	0.001-0.25	0.976	93 (12)	83 (8)	92 (7)	106 (11)	113 (8)
**Penthiopyrad**	0.001	0.001-0.25	0.989	97 (5)	99 (11)	79 (13)	96 (10)	112 (7)
**Permethrin (sum of isomers)**	0.002	0.002-0.25	0.981	-	81 (6)	82 (14)	87 (16)	85 (12)
**Phenylphenol, 2-**	0.001	0.001-0.25	0.965	72 (9)	107 (11)	105 (19)	73 (6)	113 (9)
**Phosalone**	0.001	0.001-0.25	0.987	77 (10)	105 (6)	93 (6)	107 (9)	96 (6)
**Phosmet**	0.001	0.001-0.25	1.000	106 (6)	100 (7)	95 (11)	86 (10)	87 (8)
**Phoxim**	0.001	0.001-0.25	0.999	105 (14)	92 (7)	83 (9)	104 (10)	92 (11)
**Piperonyl butoxide**	0.005	0.005-0.25	0.985	-	-	79 (13)	77 (7)	79 (3)
**Pirimicarb**	0.001	0.001-0.25	0.963	106 (19)	93 (10)	103 (6)	92 (7)	86 (6)
**Pirimicarb-desmethyl**	0.001	0.001-0.25	0.985	108 (18)	115 (7)	107 (4)	93 (4)	92 (10)
**Pirimiphos-ethyl**	0.001	0.001-0.25	0.989	85 (5)	90 (6)	91 (7)	105 (14)	118 (13)
**Pirimiphos-methyl**	0.001	0.001-0.25	0.984	119 (10)	87 (7)	94 (3)	107 (18)	107 (2)
**Prochloraz**	0.001	0.001-0.25	0.986	116 (12)	77 (16)	106 (11)	87 (5)	97 (4)
**Procymidone**	0.001	0.001-0.25	0.981	80 (14)	97 (7)	103 (11)	84 (4)	84 (6)
**Profenofos**	0.001	0.001-0.25	0.993	72 (12)	93 (6)	91 (5)	82 (14)	103 (6)
**Propargite**	0.001	0.001-0.25	0.996	96 (19)	82 (7)	90 (13)	86 (7)	88 (3)
**Propetamphos**	0.001	0.001-0.25	0.987	86 (5)	105 (5)	96 (9)	93 (8)	115 (4)
**Propyzamide**	0.001	0.001-0.25	0.996	74 (10)	101 (6)	72 (11)	95 (15)	102 (13)
**Prosulfocarb**	0.001	0.001-0.25	0.981	83 (12)	92 (6)	87 (14)	94 (16)	99 (10)
**Prothioconazole-desthio**	0.005	0.005-0.25	0.976	-	-	81 (6)	114 (5)	93 (10)
**Pyraclostrobin**	0.001	0.001-0.25	1.000	99 (12)	105 (13)	96 (8)	90 (7)	109 (10)
**Pyrazophos**	0.001	0.001-0.25	0.983	95 (10)	95 (5)	92 (11)	97 (13)	106 (2)
**Pyrimethanil**	0.001	0.001-0.25	0.992	86 (3)	107 (7)	107 (12)	78 (5)	81 (5)
**Quinalphos**	0.001	0.001-0.25	0.979	87 (5)	82 (14)	81 (4)	79 (3)	72 (6)
**Quintozen**	0.001	0.001-0.25	0.990	96 (3)	93 (11)	88 (7)	80 (6)	96 (4)
**Resmethrin**	0.005	0.005-0.25	0.986	-	-	102 (18)	115 (4)	117 (4)
**Spinosyn-A**	0.005	0.005-0.25	0.987	-	-	95 (5)	92 (6)	96 (7)
**Spinosyn-D**	0.005	0.005-0.25	0.983	-	-	109 (10)	83 (7)	94 (3)
**Spiroxamine**	0.001	0.001-0.25	0.998	99 (18)	92 (5)	96 (8)	106 (7)	108 (3)
**Sulfoxaflor**	0.002	0.002-0.25	0.996	-	85 (6)	94 (10)	92 (12)	116 (2)
**Tebuconazole**	0.001	0.001-0.25	0.994	90 (13)	83 (6)	82 (7)	86 (4)	96 (11)
**Tebufenozide**	0.001	0.001-0.25	0.997	105 (7)	87 (4)	96 (4)	91 (10)	93 (9)
**Tebufenpyrad**	0.001	0.001-0.25	0.988	96 (6)	96 (7)	115 (14)	108 (13)	92 (3)
**Tecnazene**	0.001	0.001-0.25	0.984	79 (7)	95 (3)	117 (8)	102 (2)	91 (14)
**Terbufos**	0.001	0.001-0.25	0.979	72 (16)	116 (11)	87 (5)	101 (4)	97 (9)
**Terbufos-sulfone**	0.002	0.002-0.25	0.964	-	104 (9)	97 (14)	79 (8)	70 (10)
**Terbufos-sulfoxide**	0.002	0.002-0.25	0.971	-	82 (15)	95 (6)	93 (12)	87 (14)
**Terbuthylazine**	0.001	0.001-0.25	0.997	80 (12)	83 (9)	104 (10)	98 (6)	99 (7)
**Tetrachlorvinphos**	0.001	0.001-0.25	0.991	102 (18)	105 (4)	109 (5)	86 (11)	105 (6)
**Tetraconazole**	0.001	0.001-0.25	0.997	103 (12)	108 (7)	95 (15)	95 (8)	90 (10)
**Tetramethrin**	0.001	0.001-0.25	0.992	86 (15)	88 (5)	101 (9)	106 (8)	102 (4)
**Thiacloprid**	0.001	0.001-0.25	0.986	77 (17)	80 (6)	79 (8)	81 (10)	100 (5)
**Thiamethoxam**	0.001	0.001-0.25	0.984	86 (16)	91 (6)	94 (14)	87 (16)	75 (12)
**Thiophanate-methyl**	0.001	0.001-0.25	0.989	94 (10)	77 (11)	103 (5)	94 (6)	103 (9)
**Tolclofos-methyl**	0.001	0.001-0.25	0.974	92 (15)	87 (11)	96 (4)	92 (6)	92 (7)
**Triazophos**	0.001	0.001-0.25	0.989	91 (10)	81 (6)	92 (5)	74 (11)	87 (6)
**Trichlorfon**	0.001	0.001-0.25	0.999	95 (8)	84 (11)	105 (8)	113 (12)	96 (8)
**Trifloxystrobin**	0.001	0.001-0.25	0.993	85 (12)	74 (10)	118 (7)	97 (5)	921 (11)

**Trifluralin**	0.001	0.001-0.25	0.995	93 (4)	71 (13)	115 (16)	117 (7)	101 (12)
**Vinclozolin**	0.001	0.001-0.25	0.982	84 (3)	82 (3)	112 (4)	107 (10)	98 (10)
				0.025 mg/kg	***0.04 mg/kg***	***0.05 mg/kg***	***0.1 mg/kg***	***0.2 mg/kg***
**Glufosinate**	0.025	0.025-0.2	0.985	104 (12)	92 (11)	103 (10)	95 (5)	101 (6)
**Glyphosate**	0.025	0.025-0.2	0.993	92 (14)	112 (18)	108 (17)	91 (8)	96 (6)
***N*-Acetyl AMPA**	0.025	0.025-0.2	0.996	84 (13)	108 (19)	96 (15)	102 (8)	106 (9)

Table S4. Validation results for metal determination in honey by ICP-MS

Element	LOD (mg/kg)	LOQ (mg/kg)	Linearity range (mg/kg)	R^2	Precision (RSD_R %)	Recovery (%)
**Al**	0.008	0.025	0-0.1	0.995	7.25	95
**V**	0.009	0.027	0-0.1	0.999	5.52	91
**Cr**	0.005	0.016	0-0.1	0.999	4.01	92
**Mn**	0.007	0.020	0-0.1	0.998	2.53	95
**Fe**	0.005	0.005	0-10	0.999	3.22	102
**Co**	0.004	0.012	0-0.1	0.997	4.42	95
**Ni**	0.003	0.019	0-0.1	0.999	4.63	98
**Cu**	0.003	0.010	0-0.1	0.999	6.23	94
**Zn**	0.006	0.017	0-0.1	0.999	1.95	96
**As**	0.005	0.015	0-0.1	0.999	1.40	98
**Se**	0.009	0.028	0-0.1	0.999	3.86	99
**Mo**	0.006	0.018	0-0.1	0.999	5.69	97
**Ag**	0.004	0.011	0-0.1	0.998	2.24	95
**Cd**	0.003	0.010	0-0.1	0.999	2.27	99
**Ba**	0.003	0.010	0-0.1	0.999	4.45	91
**Pb**	0.003	0.010	0-0.1	0.999	2.89	98
**Hg**	0.0003	0.001	0-5	0.997	6.38	97

***specificity:** the slopes of the calibration curves obtained in honey samples and in 5% HNO_3 were compared, and the coefficient of variation (CV) between 5% HNO_3 and the sample matrix was below 10%, indicating that no significant matrix effect was present.

Table S5. Optimized instrumental conditions for ICP-MS Agilent 7900

**Nebulizer**	MicroMist	**Peak pattern**	1 point	
**Spray chamber**	Cyclonic	**Replicates**	3	
**Interface**	Pt cones	**Sweep/replicate**	100	
**Mass analyser**	Quadropole	**Sample uptake rate**	0.4 rps	
**RF power**	1.55 kW	**Stabilization time**	No gas	He
**Acq mode**	Spectrum		10 s	5 s
**Ion lenses mode**	x-Lens	**Plasma gas**	14.99 L/min	
**Tune parameters**	Standard tune	**Aux gas**	0.9 L/min	
**Carrier gas**	1.07 L/min	**Omega Bias**	-80 V	
		**Omega Lens**	8.4 V	

Table S6. Pesticides identified in honey samples and their concentrations obtained.

Sample code	Acetamiprid	Coumaphos	DMF	DMPF	Thiacloprid	Glyphosate
**1 HONEY 1**	<LOQ	0.022	0.003	0.003	<LOQ	<LOQ
**2 HONEY 1**	0.004	0.010	0.004	0.002	<LOQ	<LOQ
**3 HONEY 1**	<LOQ	0.005	0.003	0.005	<LOQ	<LOQ
**4 HONEY 1**	<LOQ	0.002	0.004	<LOQ	<LOQ	<LOQ
**5 HONEY 1**	<LOQ	0.002	0.033	<LOQ	<LOQ	<LOQ
**6 HONEY 1**	0.002	0.005	0.008	0.004	<LOQ	<LOQ
**7 HONEY 2**	0.004	0.006	0.005	0.009	<LOQ	<LOQ
**7 HONEY 3**	0.003	0.005	0.004	0.007	<LOQ	<LOQ
**7 HONEY 4**	0.002	0.005	0.005	0.008	<LOQ	<LOQ
**8 HONEY 1**	<LOQ	0.004	0.028	0.028	<LOQ	<LOQ
**9 HONEY 1**	<LOQ	0.039	0.004	0.003	<LOQ	<LOQ
**10 HONEY 1**	<LOQ	0.020	<LOQ	<LOQ	<LOQ	<LOQ
**12 HONEY 1**	<LOQ	0.007	<LOQ	<LOQ	<LOQ	<LOQ
**13 HONEY 1**	0.001	0.005	0.027	0.012	0.001	<LOQ
**14 HONEY 1**	<LOQ	0.002	0.002	<LOQ	<LOQ	<LOQ
**15 HONEY 1**	<LOQ	0.003	0.006	0.005	<LOQ	<LOQ
**16 HONEY 1**	<LOQ	0.017	0.008	0.014	<LOQ	<LOQ
**17 HONEY 1**	<LOQ	0.004	<LOQ	<LOQ	<LOQ	<LOQ
**18 HONEY 1**	<LOQ	<LOQ	0.004	0.004	<LOQ	<LOQ
**19 HONEY 1**	<LOQ	<LOQ	0.004	0.004	<LOQ	**0.076**
**20 HONEY 1**	<LOQ	0.001	0.009	0.012	<LOQ	**0.056**
**21 HONEY 1**	<LOQ	0.007	0.003	<LOQ	<LOQ	**0.066**
**LOQ**	0.001	0.001	0.005		0.001	0.025
**MRL for honey**	0.05*	0.1	0.2<br>as sum of amitraz and its metabolites containing the 2.4-dimethylaniline moiety		0.2	0.05*

*MRL is set at a "default LOQ"; ** all values expressed as mg/kg; ***values in bold indicate exceedance of the MRL for honey

Table S7. Pesticides identified in comb honey samples and their concentrations obtained.

Sample code	Acetamiprid	Azoxystrobin	Boscalid	Coumaphos	Cyprodinil	Dimoxystrobin	Fipronil-sulfone	Fluvalinate. tau-	Fluopyram	Fluxapyroxad
**1 COMB HONEY 1**	0.002	<LOQ	<LOQ	**0.292**	0.004	<LOQ	**0.019**	<LOQ	<LOQ	<LOQ
**1 COMB HONEY 2**	<LOQ	<LOQ	<LOQ	**0.999**	<LOQ	<LOQ	**0.014**	0.004	<LOQ	<LOQ
**2 COMB HONEY 1**	0.010	0.001	0.002	**0.176**	<LOQ	0.004	**0.009**	<LOQ	<LOQ	<LOQ
**2 COMB HONEY 2**	0.002	<LOQ	0.004	**0.541**	<LOQ	0.006	**0.024**	0.004	<LOQ	<LOQ
**2 COMB HONEY 3**	<LOQ	<LOQ	<LOQ	**0.179**	0.004	<LOQ	**0.015**	<LOQ	<LOQ	<LOQ
**3 COMB HONEY 1**	<LOQ	<LOQ	<LOQ	0.085	<LOQ	<LOQ	**0.006**	<LOQ	<LOQ	<LOQ
**3 COMB HONEY 2**	<LOQ	<LOQ	<LOQ	**0.364**	<LOQ	<LOQ	**0.012**	0.003	<LOQ	<LOQ
**4 COMB HONEY 1**	<LOQ	<LOQ	<LOQ	0.040	<LOQ	<LOQ	**0.013**	<LOQ	<LOQ	<LOQ
**5 COMB HONEY 1**	<LOQ	<LOQ	<LOQ	0.033	<LOQ	<LOQ	**0.012**	<LOQ	<LOQ	<LOQ
**6 COMB HONEY 1**	0.037	<LOQ	0.003	**0.257**	<LOQ	0.003	**0.019**	0.002	<LOQ	<LOQ
**6 COMB HONEY 2**	0.011	<LOQ	<LOQ	0.036	<LOQ	<LOQ	**0.012**	<LOQ	<LOQ	<LOQ
**7 COMB HONEY 1**	0.005	<LOQ	<LOQ	**0.189**	<LOQ	<LOQ	**0.024**	0.003	<LOQ	<LOQ
**8 COMB HONEY 1**	<LOQ	<LOQ	<LOQ	0.059	<LOQ	<LOQ	**0.047**	<LOQ	<LOQ	<LOQ
**9 COMB HONEY 1**	<LOQ	0.001	<LOQ	**0.486**	<LOQ	<LOQ	**0.035**	<LOQ	0.003	<LOQ
**9 COMB HONEY 2**	<LOQ	<LOQ	<LOQ	**0.156**	<LOQ	<LOQ	**0.017**	<LOQ	<LOQ	<LOQ
**9 COMB HONEY 3**	<LOQ	<LOQ	<LOQ	0.081	<LOQ	<LOQ	**0.022**	<LOQ	<LOQ	<LOQ
**10 COMB HONEY 1**	0.001	0.002	<LOQ	**0.405**	<LOQ	<LOQ	**0.038**	0.002	<LOQ	0.001
**10 COMB HONEY 2**	<LOQ	<LOQ	<LOQ	**0.476**	<LOQ	<LOQ	**0.015**	0.002	<LOQ	0.002
**10 COMB HONEY 3**	<LOQ	<LOQ	<LOQ	**0.125**	<LOQ	<LOQ	**0.012**	<LOQ	<LOQ	<LOQ
**12 COMB HONEY 1**	<LOQ	<LOQ	<LOQ	0.077	<LOQ	<LOQ	**0.016**	<LOQ	<LOQ	<LOQ
**12 COMB HONEY 2**	<LOQ	<LOQ	<LOQ	**0.291**	<LOQ	<LOQ	**0.018**	<LOQ	<LOQ	<LOQ
**13 COMB HONEY 1**	<LOQ	<LOQ	<LOQ	0.067	<LOQ	<LOQ	**0.026**	<LOQ	<LOQ	<LOQ
**14 COMB HONEY 1**	<LOQ	<LOQ	0.002	0.036	0.004	0.001	**0.019**	<LOQ	0.002	<LOQ
**15 COMB HONEY 1**	<LOQ	<LOQ	<LOQ	0.011	<LOQ	<LOQ	**0.030**	<LOQ	<LOQ	<LOQ
**15 COMB HONEY 2**	<LOQ	<LOQ	<LOQ	0.034	<LOQ	<LOQ	**0.034**	<LOQ	<LOQ	<LOQ
**15 COMB HONEY 3**	<LOQ	<LOQ	<LOQ	0.044	<LOQ	0.001	**0.032**	<LOQ	<LOQ	<LOQ
**16 COMB HONEY 4**	<LOQ	<LOQ	<LOQ	**0.511**	<LOQ	<LOQ	**0.056**	0.020	<LOQ	0.001
**16 COMB HONEY 18**	<LOQ	<LOQ	<LOQ	0.086	<LOQ	<LOQ	**0.010**	<LOQ	<LOQ	<LOQ
**16 COMB HONEY 36**	<LOQ	0.001	<LOQ	**0.292**	<LOQ	<LOQ	**0.059**	0.002	<LOQ	0.002
**17 COMB HONEY 2**	<LOQ	<LOQ	<LOQ	0.028	<LOQ	<LOQ	**0.019**	<LOQ	<LOQ	<LOQ
**17 COMB HONEY 22**	<LOQ	<LOQ	<LOQ	0.073	<LOQ	<LOQ	**0.024**	<LOQ	<LOQ	<LOQ
**17 COMB HONEY 33**	<LOQ	<LOQ	<LOQ	**0.277**	<LOQ	<LOQ	**0.015**	0.006	<LOQ	<LOQ
**18 COMB HONEY 1**	<LOQ	<LOQ	<LOQ	0.018	<LOQ	<LOQ	**0.046**	0.005	<LOQ	<LOQ
**20 COMB HONEY 1**	<LOQ	<LOQ	<LOQ	**0.924**	<LOQ	<LOQ	**0.016**	<LOQ	<LOQ	<LOQ

**20 COMB HONEY 2**	<LOQ	<LOQ	<LOQ	**1.90**	<LOQ	<LOQ	**0.061**	0.008	<LOQ	<LOQ
**21 COMB HONEY 1**	<LOQ	<LOQ	<LOQ	0.069	<LOQ	<LOQ	**0.027**	<LOQ	<LOQ	<LOQ
**21 COMB HONEY 2**	<LOQ	<LOQ	<LOQ	0.016	<LOQ	<LOQ	**0.008**	<LOQ	<LOQ	<LOQ
**21 COMB HONEY 3**	<LOQ	<LOQ	<LOQ	0.053	<LOQ	<LOQ	**0.021**	0.004	<LOQ	<LOQ
**LOQ**	0.001	0.001	0.001	0.001	0.001	0.001	0.001	0.002	0.001	0.001
**MRL for honey**	0.05*	0.05*	0.15	0.1	0.05*	0.05*	0.005*	0.05*	0.05*	0.05*
**Sample code**	**Permetrin (sum)**	**Pyrimethanil**	**DMF**	**DMPF**	**Propargite**	**Pyraclostrobin**	**Trifloxystrobin**	**Glyphosate**	***N*-Acetyl AMPA**	
**1 COMB HONEY 1**	0.002	<LOQ	0.019	<LOQ	<LOQ	<LOQ	0.016	<LOQ	<LOQ	
**1 COMB HONEY 2**	0.003	<LOQ	0.008	<LOQ	<LOQ	<LOQ	0.015	<LOQ	<LOQ	
**2 COMB HONEY 1**	0.003	<LOQ	<LOQ	<LOQ	<LOQ	<LOQ	0.015	<LOQ	<LOQ	
**2 COMB HONEY 2**	<LOQ	<LOQ	0.006	0.019	<LOQ	<LOQ	0.014	<LOQ	<LOQ	
**2 COMB HONEY 3**	<LOQ	<LOQ	<LOQ	<LOQ	<LOQ	<LOQ	0.013	<LOQ	<LOQ	
**3 COMB HONEY 1**	<LOQ	<LOQ	<LOQ	0.011	<LOQ	<LOQ	0.025	<LOQ	<LOQ	
**3 COMB HONEY 2**	0.002	<LOQ	<LOQ	0.016	0.012	<LOQ	0.019	<LOQ	<LOQ	
**4 COMB HONEY 1**	<LOQ	<LOQ	<LOQ	<LOQ	<LOQ	<LOQ	0.021	<LOQ	<LOQ	
**5 COMB HONEY 1**	<LOQ	<LOQ	0.015	<LOQ	<LOQ	<LOQ	0.028	<LOQ	<LOQ	
**6 COMB HONEY 1**	<LOQ	<LOQ	0.008	0.007	<LOQ	<LOQ	0.021	<LOQ	<LOQ	
**6 COMB HONEY 2**	<LOQ	<LOQ	0.009	0.013	<LOQ	<LOQ	0.024	<LOQ	<LOQ	
**7 COMB HONEY 1**	<LOQ	<LOQ	0.008	0.020	<LOQ	<LOQ	0.030	<LOQ	0.019	
**8 COMB HONEY 1**	<LOQ	<LOQ	0.007	0.015	<LOQ	<LOQ	0.031	<LOQ	<LOQ	
**9 COMB HONEY 1**	<LOQ	<LOQ	<LOQ	<LOQ	<LOQ	<LOQ	0.026	<LOQ	<LOQ	
**9 COMB HONEY 2**	0.005	<LOQ	<LOQ	<LOQ	<LOQ	<LOQ	0.032	<LOQ	<LOQ	
**9 COMB HONEY 3**	<LOQ	<LOQ	<LOQ	<LOQ	<LOQ	<LOQ	0.034	<LOQ	<LOQ	
**10 COMB HONEY 1**	<LOQ	<LOQ	<LOQ	<LOQ	<LOQ	0.002	0.035	<LOQ	<LOQ	
**10 COMB HONEY 2**	<LOQ	<LOQ	<LOQ	0.017	<LOQ	<LOQ	0.034	<LOQ	<LOQ	
**10 COMB HONEY 3**	<LOQ	<LOQ	<LOQ	<LOQ	<LOQ	<LOQ	0.035	<LOQ	<LOQ	
**12 COMB HONEY 1**	<LOQ	<LOQ	<LOQ	<LOQ	<LOQ	<LOQ	0.038	<LOQ	<LOQ	
**12 COMB HONEY 2**	<LOQ	<LOQ	<LOQ	<LOQ	<LOQ	<LOQ	0.028	<LOQ	<LOQ	
**13 COMB HONEY 1**	<LOQ	<LOQ	0.048	0.045	<LOQ	<LOQ	0.030	<LOQ	0.020	
**14 COMB HONEY 1**	<LOQ	0.001	<LOQ	<LOQ	<LOQ	<LOQ	0.042	<LOQ	<LOQ	
**15 COMB HONEY 1**	<LOQ	<LOQ	<LOQ	0.009	<LOQ	<LOQ	0.064	<LOQ	<LOQ	
**15 COMB HONEY 2**	<LOQ	<LOQ	0.005	0.012	<LOQ	<LOQ	0.054	<LOQ	<LOQ	
**15 COMB HONEY 3**	<LOQ	<LOQ	<LOQ	0.006	<LOQ	<LOQ	0.052	<LOQ	<LOQ	
**16 COMB HONEY 4**	<LOQ	<LOQ	0.023	**0.256**	<LOQ	<LOQ	0.009	<LOQ	<LOQ	
**16 COMB HONEY 18**	<LOQ	<LOQ	<LOQ	0.014	<LOQ	<LOQ	0.056	<LOQ	<LOQ	
**16 COMB HONEY 36**	<LOQ	<LOQ	**0.030**	**0.132**	<LOQ	<LOQ	0.060	<LOQ	<LOQ	
**17 COMB HONEY 2**	<LOQ	<LOQ	<LOQ	<LOQ	0.005	<LOQ	**0.083**	<LOQ	<LOQ	

**17 COMB HONEY 22**	<LOQ	<LOQ	<LOQ	<LOQ	0.001	<LOQ	**0.091**	<LOQ	<LOQ
**17 COMB HONEY 33**	<LOQ	<LOQ	<LOQ	<LOQ	0.015	<LOQ	**0.071**	<LOQ	<LOQ
**18 COMB HONEY 1**	<LOQ	<LOQ	0.006	0.011	0.002	<LOQ	0.029	<LOQ	<LOQ
**20 COMB HONEY 1**	<LOQ	<LOQ	<LOQ	0.007	<LOQ	<LOQ	**0.071**	<LOQ	<LOQ
**20 COMB HONEY 2**	<LOQ	<LOQ	<LOQ	0.012	<LOQ	<LOQ	**0.074**	<LOQ	<LOQ
**21 COMB HONEY 1**	<LOQ	<LOQ	<LOQ	0.020	<LOQ	<LOQ	0.062	0.044	<LOQ
**21 COMB HONEY 2**	<LOQ	<LOQ	<LOQ	<LOQ	0.002	<LOQ	0.060	0.037	<LOQ
**21 COMB HONEY 3**	<LOQ	<LOQ	0.008	0.048	0.005	<LOQ	0.075	**0.194**	<LOQ
**LOQ**	0.002	0.001	0.005		0.001	0.001	0.001	0.025	0.025
**MRL for honey**	0.3	0.05*	0.2 (as sum of amitraz and its metabolites containing the 2.4-dimethylaniline moiety)		0.05*	0.05*	0.07	0.05*	

*MRL is set at a “default LOQ”; ** all values expressed as mg/kg; ***values in bold indicate exceedance of the MRL for honey
